# Supplementary material for: CanDIG: Federated network across Canada for multi-omic and health data discovery and analysis
Source: Cell Genom. 2021 Nov 10;1(2):100033. doi: 10.1016/j.xgen.2021.100033 (PMC9903648; doi:10.1016/j.xgen.2021.100033)
Supplement: Document S1. Figures S1–S3 and Tables S1–S3 [file mmc1.pdf]

Cell Genomics, Volume *1*

## Supplemental information

# CanDIG: Federated network across Canada for multi-omic and health data discovery and analysis

**L. Jonathan Dursi, Zoltan Bozoky, Richard de Borja, Haoyuan Li, David Bujold, Adam Lipski, Shaikh Farhan Rashid, Amanjeev Sethi, Neelam Memon, Dashaylan Naidoo, Felipe Coral-Sasso, Matthew Wong, P-O Quirion, Zhibin Lu, Samarth Agarwal, Yuriy Pavlov, Andrew Ponomarev, Mia Husic, Krista Pace, Samantha Palmer, Stephanie A. Grover, Sevan Hakgor, Lillian L. Siu, David Malkin, Carl Virtanen, Trevor J. Pugh, Pierre-Étienne Jacques, Yann Joly, Steven J.M. Jones, Guillaume Bourque, and Michael Brudno**

## Supplemental Information for CanDIG: Federated Network across Canada for multi-omic and health data discovery and analysis

### Supplemental Notes

Section 1: CanDIG Implementation Approach
Section 2: A Granular Model of Data Federations
Section 3: Roles and Responsibilities in the CanDIG model
Section 4: Local Differential Privacy Proof of Concept

### Supplemental Figures

Figure S1: Current and Coming Use of GA4GH standards in the CanDIG Platform
Figure S2: Division of Roles and Responsibilities to CanDIG Federation Stakeholders
Figure S3: Proof of Concept Implementation of Local Differential Privacy

### Supplemental Tables

Table S1: CanDIG Datasets
Table S2: Six-Dimension Approach to Designing Data Federations
Table S3: Roles and Responsibilities of CanDIG Partners

## Supplemental Notes

### Section 1: CanDIG Implementation Approach

The CanDIG effort decided on several principles before building anything.

CanDIG:

- Is **fully distributed**: All data, and all infrastructure, is completely distributed; no shared or centralized services. All coordination is done at the level of policy, protocol, or software development.
- **Gives the sites full local control** of authorization to data: Consistent with common governance and policies, local data custodians have complete control over access to their data, and auditability/observability into data access and use. We do not support large-scale data transfers and downloads as they sacrifice later auditability.
- Is **API-first**: Since we are building a platform whose success hinges on the interaction between users and multiple sites, new development will rely on API-first design, with APIs developed and documented, and services and clients built on this. This ensures documentation of the APIs, interoperability between clients, and alignment with GA4GH efforts.
- **Supports private consented research data**: Our mission is to connect privacy-sensitive, although not directly identifying, human health data. We will rely on modern authentication and authorization technologies to that end, and follow GA4GH Security Working Group's best practices. Since CanDIG is also fully distributed there is no central infrastructure to maintain or secure. We don't intend to support non-private data (e.g., from model organisms) or unconsented data from routine clinical care.
- Is **Open Source, Standards-Based, and Interoperable**: CanDIG builds on existing standards, on matters both technical and genomic (via its role as a driver project for the GA4GH effort). We enthusiastically adopt software written elsewhere. This approach allows interoperability as wide as possible while focusing our efforts only where it addresses our particular needs.

CanDIG-server (web resources) is a core part of the v1 platform, implementing many of the APIs it supports (and all that are mandatory for a v1 node). In the CanDIG server, many API endpoints are built on the retired GA4GH reference implementation (GA4GH Server: web resources) – indeed, roughly half of the code in the CanDIG-server comes from the reference implementation – and thus inherits its style, and how they were implemented. HTTP RPC-style methods are used for data retrieval, where the POST endpoints have a pre-defined request and response structure in protobuf, whose JSON form is accepted. We've built on that to allow binary protobuf messages between sites where the size and serializability of the response is an issue. In early work, we added 23 sets of newly-added clinical and pipeline metadata endpoints, each permitting both a simple request and a complex search with filtering. We support a number of operators in the filter objects, including lt, le, eq, ne, ge, gt, contains, in, etc. To ensure the ease of use of our APIs, we provide a Swagger API documentation, whose definition is widely used and can be visualized via a number of tools. We also provide a number of sample queries

that cover most of the common use-cases. By implementing these searches consistently across services we enabled complex cross-datatype queries; future work will investigate the new standard GA4GH Data Connect for such searches. In addition, we added a clinical and phenotypic data model informed by rare-disease work and a late pre-mCODE standard for cancer studies[1].

Having an initial set of APIs over genomic and relevant clinical data allowed us to bootstrap our federation and distributed authentication and authorization infrastructure atop. In our decentralized federation, there is no central “CanDIG” identity; each user has a home site, typically the institution at which they work, where they can log in using their local credentials and can view the dashboard there. This propagates the queries necessary to drive the dashboard across to all federation partners. Currently, a simple, single-step fan-out is sufficient. Each site in the federation recognizes the identity of the other sites and makes authorization decisions regarding access to the data it hosts. The granularity of that authorization allows for multiple projects to be supported without exposing data from one project to researchers of another unless so authorized. That information is then presented to the user via their home site. Because the user is necessarily authorized to see the data from the response, and the home site is effectively the user’s work computer, the requests can be safely aggregated at the home sites. Other topologies and communication strategies have been tested and will be used as the number of our participating sites grows.

Key to our authentication is open-source software components: Keycloak and Tyk, one of which exists at each site. Managing the Keycloak instance is a shared responsibility between the CanDIG developers and the local host site IT team; it allows users to log in using research institution or hospital credentials, allowing trusted federation partners to handle identity management, while providing OpenID Connect identity tokens. Keycloak is connected to each site’s internal authentication service and does not store any credentials itself. Keycloak has out-of-the-box support for active directory, LDAP, and Kerberos for internal network user registries. We do not federate identities; we recognize as part of the federation agreement the distributed identities supplied by the federation partners. Relying on standard tools has made it easy to extend our approach to authentication - as part of the CINECA project, we have shown that we can conditionally accept ELIXIR OIDC tokens (CINECA: web resources) as an identity token. We had to fix a bug in Keycloak (web resources) to do it, but by doing so we contribute to the entire ecosystem of users interested in using GA4GH passport tokens.

Services rely on the user's OIDC Identity Token associated with each request - after a final validation – to authorize data requests. In the CanDIG model, authorization is informed by platform-level information, but authorization decision-making is always strictly local. Currently, authorization information is maintained in a local text-based database; users with different project roles or areas of specialty are granted different "access levels" to a dataset by the study's DAC, with each record of clinical and phenotypic data belonging to a dataset and each property within the record having a default access level. However, the required access levels of each item - each property of any particular row - can be increased, allowing, for instance, data custodians responsible for data from marginalized populations to require additional levels of

authorization to access the study data, reinforcing privacy protections for individuals particularly vulnerable to medical and other discrimination.

At each site, the open-source API gateway Tyk serves as a "single sign-on" for any services behind it; it validates the OIDC tokens from any of the federation Keycloak instances, and once the request has a recognized identity token it allows rewriting or rerouting of requests enabling us to maintain a static external set of APIs while changing internals. Tyk also has essential features such as session handling, rate limiting and logging of incoming requests. We implemented middleware to also handle the "OAuth2 dance", to perform the 3-way authentication handshake involving the client and Keycloak, so that Tyk serves as the "Relying Party" for OIDC/OAuth2.

With the authorization in place, complex queries that researchers wish to perform programmatically or that are awkward to use a web interface for are available through increasingly rich APIs connecting the individual services and APIs supported by the platform. These components were unified through a single /search endpoint returning the information, or /count which returns counting aggregations, implemented in the candig-server codebase.

Once the distributed identity, local authorization, APIs, and API gateway were in place, it was straightforward to begin refactoring and adding additional capabilities. The federation component, originally built into candig-server, has been pulled out into its own service with extended functionality; this allows us to integrate new services, with one site supporting RNAGet and two supporting htsget, allowing us to both provide needed functionality early and test-driving new services and methods for integration into next versions of the software implementation.

With the federation approach now solid and battle-tested, the bootstrapping and learning approach we've taken with the development of the federation and platform are now able to more rapidly iterate. We now are actively working on version two (CanDIG V2: web resources) of the software stack, with technical write-ups underway, and have plans for version three (including a very different approach to clinical data modelling (OMOP Service: web resources), using the well-known OHDSI OMOP Common Data Model (web resources)), but crucially the basic federation approach and overall architecture will differ very little. Our implementation of authorization will grow more sophisticated in some ways (web resources), with a policy engine (OPA: web resources) simplifying the coordination of authorization across multiple services and addition of DAC portals using ELIXIR's REMS (web resources) tool; and less complicated in others – while the per-field granularity of v1's authorization was desired by some data custodians, in practice it was rarely used and will be handled more simply. But crucially the basic approach of local authorization informed by federation-level information remains the same, and only the box names on an architecture diagram change.

What we've learned and are iterating on also includes operations. We've gained experience working with local site managers on shared tools that cross the CanDIG/site service boundaries with Keycloak. Work underway for the next version which will support larger scale and

automation, we have defined new boundaries. MinIO and GA4GH Data Registry Service (DRS), already implemented at one site, mark the standard APIs for storage which the CanDIG stack can leverage but allow sites to use a variety of back-end storage systems. Similarly, the GA4GH Workflow Execution Service (WES) (web resources) and the Common Workflow Language (CWL)[2] implemented at two but not yet exposed to the user allow us to decouple the definition and invocation of computational pipelines from how they are run on back-end computational infrastructure.

To support standards and interoperability, we build wherever possible on existing technical and genomic standards. As mentioned earlier, we use OIDC for authentication, and OpenAPI for defining APIs; we rely on standard genomics formats like CRAM and VCF; we use and help shape GA4GH APIs such as Htsget, Beacon, and RNAget, GA4GH ontologies such as DUO for consented use for data. Upcoming work will use standards in exposing services such as CWL, GA4GH WES, and DRS. We have established the success of this approach in promoting interoperability by demonstrating initial two-way authentication with the ELIXIR Authentication and Authorization Infrastructure[3].

## Section 2: A Granular Model of Data Federations

We have employed a six-dimension approach to describing the design of data federations, using the distinctions outlined below (Supplementary Table 2). Foundationally, a governance model which includes how federation peers join and leave, and the trust model required between them; how authentication of federation users work; the granularity of authorization; what queries are enabled; how queries flow through the federation; and how intermediate data is combined to be presented to the researcher.

In MME, researcher identities are only meaningful at each node, and the nodes themselves make requests of each other.

The depth of access to data often reflects deeper cooperation and shared governance between the data sites. Federations can be quite open, allowing new peers readily, such as the Beacon Network, or closed, allowing deeper access to the data or access to sensitive data but requiring formal agreements to be signed to join (such as Datashield, with deep access to data, or MME with access to deeply phenotyped childhood rare disease data).

We have a strong trust model between our participating institutions. They are all teaching hospitals and research institutions with long histories of collaboration and experience signing and honouring agreements with each other. A primary use case for CanDIG is to support national projects that the sites are collaborating on. While we do not expose raw data between sites, this strong level of trust gives us greater flexibility in the release of and combination of intermediate results in analyses.

Platform authentication is performed at the institution level – each institution provides a strong identity for its CanDIG users, and each user must have a CanDIG institution vouching for them. All requests in the platform are tied to a single user, ensuring enforceable accountability at the level of the researcher and the institutions. For authentication, we use the web—standard OpenID Connect (OIDC)[4] technology.

For controlled access data, access authorization decisions are made locally by the data sites. In our case, these sites bear ultimate responsibility for incorrectly authorized data release. However, many of the data sets stored within CanDIG are part of larger national projects which have data access committee lists maintained by one of the sites. Thus, the local authorization takes as input external data.

Query flow through our system is entirely peer-to-peer. Since every user “belongs” to an authenticating site, their queries can flow to that site, whence they propagate outwards through the closed federation to be received at peer sites. All requests through the system are associated with the single authenticated researcher who made the request; this is simplified by our adoption of OIDC authentication. While we have experimented with peer-to-peer cycle topologies to enable certain types of privacy-enhancing aggregations, for the time being, we use a simple fan-out topology.

For result aggregation, we make use of the fact that each user is strongly associated with a particular site, and so results the user is entitled to access can be safely aggregated on that site. We have also experimented with the use of homomorphic encryption to allow for global approaches to differentially private aggregations. This requires lower amounts of total noise (and thus higher result utility) to maintain privacy than our current local (or regional) differential privacy approach but is not implemented currently.

Finally, CanDIGv1 does not automate any form of platform-wide observability or auditability of access patterns across the closed federation as a whole, given the select number of sites, the closely-knit team, and the modest query rate, this has not been necessary. Approaches are being proposed for v2.

## Section 3: Roles and Responsibilities in the CanDIG model

With a “multi-tenant” platform supporting multiple research projects, and federation separating the roles of sites and the platform as a whole, the CanDIG project has had to be very explicit about the roles and responsibilities of each partner in its role (Supplementary Figure 2). Below is a more detailed listing of the roles and responsibilities outlined above (see Supplementary Table 3).

**Section 4: Local Differential Privacy Proof of Concept**

The CanDIG platform provides authorized researchers access to complex queries and analyses of distributed datasets, jointly across various supported data types. This is enabled through very fine-grained authorization. Staff and researchers with different project roles or areas of specialty are granted different "access levels" to a dataset by the study's DAC, with each record of clinical and phenotypic data belonging to a dataset and each property within the record having a default access level. However, the required access levels of each item - each property of any particular row - can be increased, allowing, for instance, data custodians responsible for data from particularly marginalized populations to require additional levels of authorization to access the study data.

With the authorization in place, complex queries that researchers wish to perform programmatically or that are awkward to use a web interface for are available through increasingly rich APIs connecting the individual services and APIs supported by the platform. These components were unified through a single /search endpoint returning the information, or /count which returns counting aggregations. These APIs supported by the platform permit researchers to programmatically perform complex queries that are not supported by the user interface. Such queries can serve both discovery and simple analyses use cases.

For custodians who are considering making data available for discovery queries to a broader range of researchers while maintaining participant privacy, CanDIG has demonstrated support for making privacy enhanced analytics queries available as counts with local differential privacy [5] as an initial proof of concept. Laplace or exponential noise can be added to counts at each site before being summed and presented to the user (Figure 1A). This can be used for data discovery, but is also enough to support complex analyses. For instance, it is possible to train ID3 machine-learning classifiers [6] to predict ancestral data from a modest number of informative SNPs in the 1000 genomes data using the existing counts functionality (ID3 Decision Tree Classifier implementation can be found here: https://github.com/CanDIG/id3-variants-training). To illustrate this we perform queries on the client side that gather counts of a small set of known-informative SNPs - a subset of 17 from a known panel of 55 [7], grouped by demographic information (the 1000 genomes ancestry), and train a decision tree classifier based on the "splits" of ancestry by presence or absence of the SNPs. ID3 decision trees work particularly well with differentially private aggregations due to their use of counts (Figure S3).

It is also possible to use the APIs to perform more traditional population genetics visualizations such as identifying SNP counts versus ancestry. Our experience with these workflows is informing the continuing development of cross-service APIs for analytics, pushing the analyses to the server rather than the client.

## Supplemental Figures

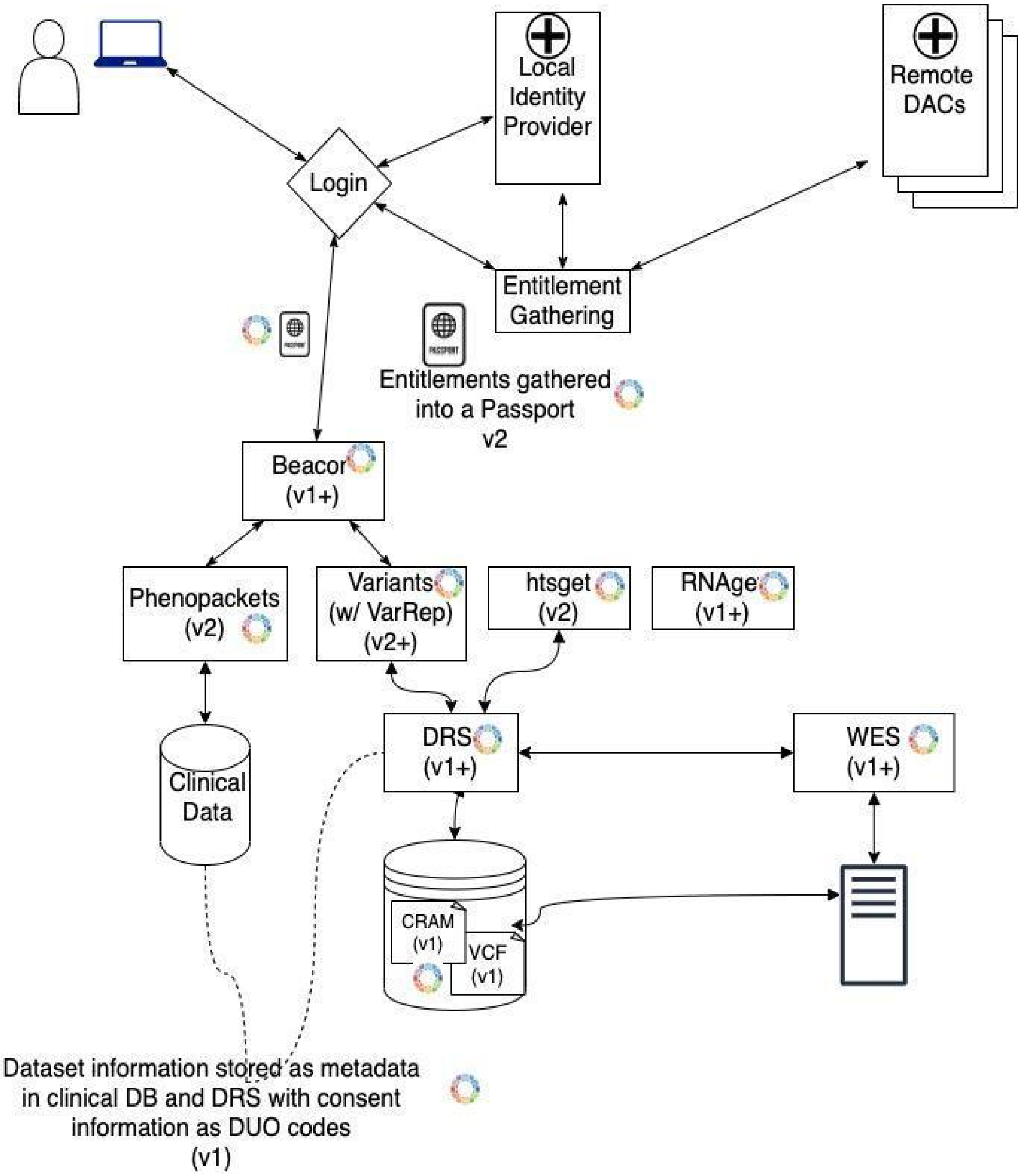


**Figure S1: Current and Coming Use of GA4GH standards in the CanDIG Platform, related to CanDIG Platform Adoption of GA4GH Standards and Technologies**. A schematic diagram illustrating how GA4GH technical standards are being used in the evolving CanDIG platform as we move towards deploying CanDIGv2. Illustrated are representations of services and standards being deployed within an individual CanDIG site, and how they build upon each other - use of standard Phenopackets eases the lightning of Beacons, a standard Variant Representation will avoid ambiguity of variants, the Data Repository Service powers Workflow Execution Service as well as other file-based services such as htsget, and internally entitlements are propagated using a representation based on Passport visas. Consent metadata uses ontologies like DUO. Not shown are services following similar patterns as others, such as RNAget or refget, and that each service identifies itself using Service-Info. Also not shown are the use of policy standards such as the Framework for Responsible Data Sharing, Breach Reporting, or Privacy and Security Policy. Tools that are a mandatory part of CanDIGv1 are noted with (v1); those that are deployed but not mandatory in v1 are noted with (v1+); those that additionally are required as part of CanDIGv2 are noted with (v2); those that are in development for future versions of v2 are noted with (v2+).

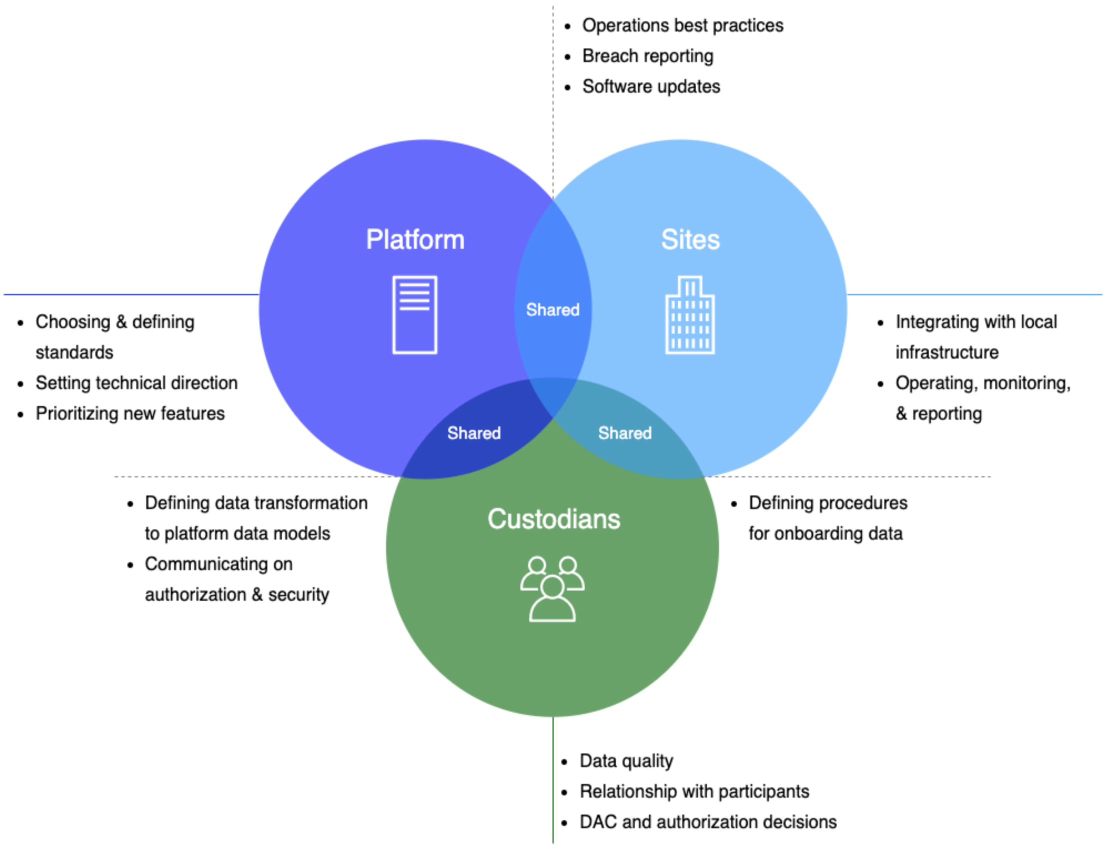


**Figure S2: Division of Roles and Responsibilities to CanDIG Federation Stakeholders, related to CanDIG Federation Governance Model**. A high-level Venn diagram of roles and responsibilities of CanDIG Platform Partners - the data custodians (such as research projects), the platform effort, and the individual sites. A more detailed list of the responsibilities of each partner is shown in Supplementary Table 3.

**A**

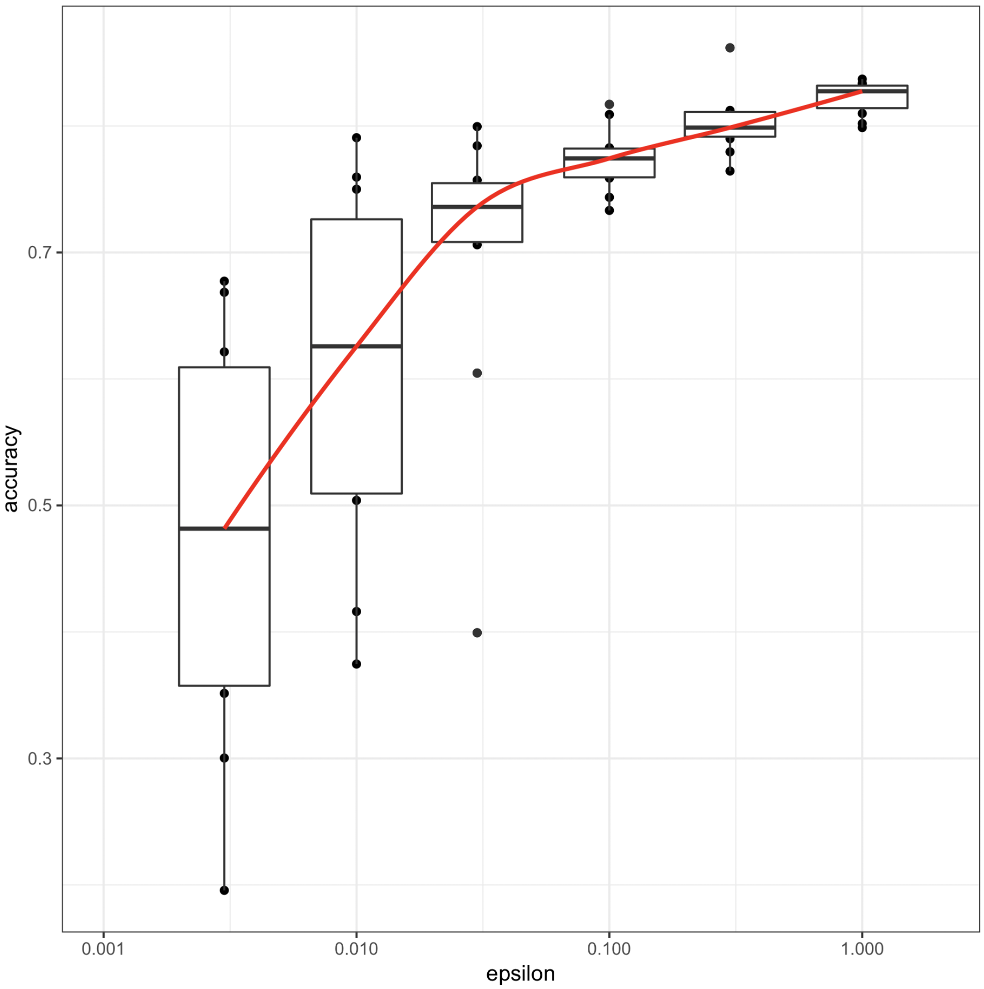


**B**

```
ID3 (label, dataset, cur_ethnicity_counts, has_variants, lacks_variants, remaining_variants, dataset)
  node ← create_node(label=label, left=∅, right=∅)

  if |remaining_variants| = 0 || argmax cur_ethnicity_counts = sum(cur_ethnicity_counts)
    node.label ← argmax cur_ethnicity_counts
    return node
  best_ig, best_v, best_ec_left, best_ec_right ← -∞, ∅, ∅, ∅
  for v in remaning_variants:
    CanDIG API call: counts_left ← count(dataset, has_variant=v ∪ has_variants, lacks_variants, by=ethnicity)
    counts_right ← clip(cur_ethnicity_counts - counts_left)
    information_gain ← gain(counts_left, counts_right)
    if information_gain > best_ig
      best_ig, best_v, best_ec_left, best_ec_right ← information_gain, v, counts_left, counts_right

  node.left ← ID3(+best_v, best_ec_left, has_variants ∪ v, lacks_variants, remaining_variants - v)
  node.right ← ID3(-best_v, best_ec_right, has_variants, lacks_variants ∪ v, remaining_variants - v)
  return node

tree ← ID3(label=root, has_variants=∅, lacks_variants=∅, remaining_variants=variants, training_dataset)
```

**Figure S3: Proof of Concept Implementation of Local Differential Privacy, related to Figure 1D**. Consistent with our approach of ensuring local control over authorization decisions, for queries or datasets where differential privacy is required we demonstrate a proof of concept implementation of the privacy enhancing analysis technique of local differential privacy. In local differential privacy, each data site determines its privacy parameter $\epsilon$ and perturbs the results with a corresponding degree of Lapacian or exponential noise. Lower $\epsilon$ means lower privacy loss, requiring more noise and lower resulting accuracy. This contrasts with global differential privacy, where a central site adds a global level of noise after getting unperturbed data from the sites. **A** Demonstrates a proof of concept, training of an ID3 tree based on 17 known ancestry informative SNPs on five of the 1000 genomes superancestries, with per-query laplacian noise (determined by the query sensitivity and $\epsilon$) added at each query, trained on 250 randomly selected training genomes and tested against held out samples. With decreasing levels of the privacy parameter $\epsilon$, privacy increases and utility is diminished; accuracy varies more widely run to run with larger amounts of noise (smaller values of $\epsilon$). Future implementations will make use of robust production-quality differential privacy implementations such as those of OpenDP (https://opendp.org/). **B** Illustrates the classification algorithm, an ID3 tree classifier, implemented on the client side.

## Supplemental Tables

| Project | Status | Number of Participants with data | Participating Sites | Genomic and other Data Types |
|---|---|---|---|---|
| Precision Oncogenomics (POG)[8] | Current | 570 | BC Cancer Canada's Michael Smith Genome Sciences Centre (BCGSC) | WGS - tumour and normal |
| INSPIRE [9–11] | Current | 106 | University Health Network (UHN) | WES - tumour and normal; RNA-Seq |
| TF4CN/COMPARISON | Current | 12 | UHN, BCGSC | WGS - tumour and normal; RNA-Seq |
| CanCOGen HostSeq (web resources) - COVID-19 Host sequencing data, initial project for CanDIGv2 | Current | 983 currently, rising to 10,000 | BCGSC, with McGill to join shortly | WGS |
| PRecision Oncology for Young peopLE (PROFYLE)[12] | In progress | 338 | McGill, SickKids, BCGSC | WGS - tumour and normal; RNA expression |
| Digital Health and Discovery Platform (2021-2025) | Upcoming | Target: 15,000 over 4 years | Initially: McGill, Centre hospitalier de l'Universite de Montreal (CHUM), UHN, BCGSC | WGS - tumour and normal, imaging, extensive clinical phenotype (mCODE) |

**Table S1: CanDIG Datasets, related to CanDIG Use By Pan-Canadian Projects**
Current and forthcoming projects supported by CanDIG, including available patient numbers and data types. CanDIG's federation and distributed authentication and authorization will be part of the DHDP (web resources), a national platform initially supporting the government of Canada and Terry Fox Research Institute-supported Marathon of Hope Cancer Centre Network (web resources), a 15,000-patient cohort-of-cohorts of consented cancer data.

| Model | Governance & Peer Trust Model | AuthN | AuthZ granularity | Queries | Query flow | Results gathering |
|---|---|---|---|---|---|---|
| Beacon Network [13] | Open | Partial recognition of external identities | Multi-dataset; open, registered access, controlled access | Single-query | Hub-spoke | Aggregation to hub |
| MME [14] | Closed | Site-to-site | Single-dataset | Single-query | Pairwise | Pairwise aggregation |
| DataShield[15] | Somewhat closed; peers trust central portal | Central identity | Fine-grained | Many (~140) primitives | Hub-spoke | Summary statistics only |
| Local EGA[16] | Closed | Central identity | Multi-dataset | File access | Hub-spoke | Aggregation to requestor |
| SPHN [17] | Closed, low-trust | Recognition of node identities | Fine-grained, multi-dataset | Multiple APIs | Peer-to-peer | Secure multi-party computation, homomorphic encryption |
| CanDIG | Closed, high-trust | Recognition of peer identities | Fine-grained, multi-dataset | Multiple APIs | Peer-to-peer | Aggregation to requesting site; optional differential privacy |

**Supplementary Table 2: Six-Dimension Approach to Designing Data Federations, related to CanDIG Federated Platform Model**
A comparison of different federation models along six dimensions - governance/peer trust, authentication, authorization granularity, query range, query flow, data gathering.

| CanDIG Platform | Data Custodians | CanDIG Sites |
|---|---|---|
| The federation of CanDIG sites, and the coordination thereof. A national steering committee of PIs, advisors, and technical leadership.<br><br>Responsible for:<br><br>● Coordinating software development (which takes place at the sites)<br>● Policy setting, including adopting GA4GH-recommendations for best practices<br>● Coordinating operating best practices, developed at the sites<br>● Road-mapping based on user (via custodians) and operational (via site) input<br>● Standards adoption and development<br>● International interoperability<br>● Working with external collaborators<br>● Onboarding new data custodians/data projects<br>● Promoting the harmonization of data access conditions (authorizations) between local custodians | May be national (with data hosted at multiple sites) or local (with data hosted at one).<br><br>Responsible for:<br><br>● Interacting with participants (consent, withdrawal)<br>● De-identification<br>● Data quality<br>● Defining authorizations, authorized users, such as through a data access committee (DAC)<br>● Defining additional requirements and feature requests | Located at a health research institution; works closely with both local data custodians and the platform effort.<br><br>Responsible for:<br><br>● Integration of the stack with local identity management, compute/storage infrastructure<br>● Providing authentication and federated query handling for local users<br>● Operating best practices, backups, monitoring<br>● Sharing knowledge and best practices<br>● Keeping the software up to date<br>● Ingesting data from local custodians<br>● Maintaining peering with federation sites<br>● Reporting<br>● Enforcing up to date authorizations<br>● Incident response and breach reporting |

**Table S3: Roles and Responsibilities of CanDIG Partners, related to CanDIG Federation Governance Model.**

## Supplementary Material Web Resources

CanDIG Server, https://github.com/candig/candig-server

GA4GH Server, https://github.com/ga4gh/ga4gh-server

CINECA, Integration of new cohort infrastructures to the ELIXIR AAI,

https://www.cineca-project.eu/blog-all/integration-of-new-cohort-infrastructures-to-the-elixir-aai

Keycloak, Pull Request, https://github.com/keycloak/keycloak/pull/7214

CanDIG V2 https://github.com/candig/candigv2

OMOP Service, https://github.com/CanDIG/omop_service

OHDSI, OMOP Common Data Model,

https://www.ohdsi.org/data-standardization/the-common-data-model/

CanDIG V2 Authentication and Authorization,

https://www.distributedgenomics.ca/posts/candigv2-aai/

Open Policy Agent (OPA), https://www.openpolicyagent.org/

ELIXIR, https://www.elixir-finland.org/en/aai-rems-2/

Workflow Execution Service (WES),

https://github.com/ga4gh/workflow-execution-service-schemas

CanCOGeN HostSeq, https://www.genomecanada.ca/en/cancogen/cancogen-hostseq

Digital Health & Discovery Platform (DHDP), https://www.dhdp.ca/

Marathon of Hope Cancer Centres, https://www.marathonofhopecancercentres.ca/

## Supplementary Material References


1. Conley, R.B., Dickson, D., Zenklusen, J.C., Al Naber, J., Messner, D.A., Atasoy, A., Chaihorsky, L., Collyar, D., Compton, C., Ferguson, M., et al. (2017). Core Clinical Data Elements for Cancer Genomic Repositories: A Multi-stakeholder Consensus. Cell *171*, 982–986.

2. Amstutz, P., Crusoe, M.R., Tijanić, N., Chapman, B., Chilton, J., Heuer, M., Kartashov, A., Leehr, D., Ménager, H., Nedeljkovich, M., et al. (2016). Common Workflow Language, v1.0.

3. Linden, M., Prochazka, M., Lappalainen, I., Bucik, D., Vyskocil, P., Kuba, M., Silén, S., Belmann, P., Sczyrba, A., Newhouse, S., et al. (2018). Common ELIXIR Service for Researcher Authentication and Authorisation. F1000Res. *7*.

4. Sakimura, N., Bradley, J., Jones, M., de Medeiros, B., and Mortimore, C. (2014). OpenID Connect Core 1.0 incorporating errata set 1. The OpenID Foundation, specification.

5. Duchi, J.C., Jordan, M.I., and Wainwright, M.J. (2013). Local Privacy and Statistical Minimax Rates. In 2013 IEEE 54th Annual Symposium on Foundations of Computer Science, pp. 429–438.

6. Quinlan, J.R. (1986). Induction of decision trees. Mach. Learn. *1*, 81–106.

7. Kidd, K.K., Speed, W.C., Pakstis, A.J., Furtado, M.R., Fang, R., Madbouly, A., Maiers, M., Middha, M., Friedlaender, F.R., and Kidd, J.R. (2014). Progress toward an efficient panel of SNPs for ancestry inference. Forensic Sci. Int. Genet. *10*, 23–32.

8. Pleasance, E., Titmuss, E., Williamson, L., Kwan, H., Culibrk, L., Zhao, E.Y., Dixon, K., Fan, K., Bowlby, R., Jones, M.R., et al. (2020). Pan-cancer analysis of advanced patient tumors reveals interactions between therapy and genomic landscapes. Nature Cancer *1*, 452–468.

9. Clouthier, D.L., Lien, S.C., Yang, S.Y.C., Nguyen, L.T., Manem, V.S.K., Gray, D., Ryczko, M., Razak, A.R.A., Lewin, J., Lheureux, S., et al. (2019). An interim report on the investigator-initiated phase 2 study of pembrolizumab immunological response evaluation (INSPIRE). J Immunother Cancer *7*, 72.

10. Bratman, S.V., Yang, S.Y.C., Iafolla, M.A.J., Liu, Z., Hansen, A.R., Bedard, P.L., Lheureux, S., Spreafico, A., Razak, A.A., Shchegrova, S., et al. (2020). Personalized circulating tumor DNA analysis as a predictive biomarker in solid tumor patients treated with pembrolizumab. Nature Cancer *1*, 873–881.

11. Cindy Yang, S.Y., Lien, S.C., Wang, B.X., Clouthier, D.L., Hanna, Y., Cirlan, I., Zhu, K., Bruce, J.P., El Ghamrasni, S., Iafolla, M.A.J., et al. (2021). Pan-cancer analysis of longitudinal metastatic tumors reveals genomic alterations and immune landscape dynamics associated with pembrolizumab sensitivity. Nat. Commun. *12*, 5137.

12. Grover, S.A., Berman, J.N., Chan, J.A., Deyell, R.J., Eisenstat, D.D., Fernandez, C.V., Grundy, P.E., Hawkins, C., Irwin, M.S., Jabado, N., et al. (2020). Terry Fox PRecision Oncology For Young peopLE (PROFYLE): A Canadian precision medicine program for

children, adolescents and young adults with hard-to-treat cancer. Cancer Res *80*, 5413.

13. Fiume, M., Cupak, M., Keenan, S., Rambla, J., de la Torre, S., Dyke, S.O.M., Brookes, A.J., Carey, K., Lloyd, D., Goodhand, P., et al. (2019). Federated discovery and sharing of genomic data using Beacons. Nat. Biotechnol. *37*, 220–224.

14. Philippakis, A.A., Azzariti, D.R., Beltran, S., Brookes, A.J., Brownstein, C.A., Brudno, M., Brunner, H.G., Buske, O.J., Carey, K., Doll, C., et al. (2015). The Matchmaker Exchange: a platform for rare disease gene discovery. Hum. Mutat. *36*, 915–921.

15. Gaye, A., Marcon, Y., Isaeva, J., LaFlamme, P., Turner, A., Jones, E.M., Minion, J., Boyd, A.W., Newby, C.J., Nuotio, M.-L., et al. (2014). DataSHIELD: taking the analysis to the data, not the data to the analysis. Int. J. Epidemiol. *43*, 1929–1944.

16. Fernández-Orth, D., Lloret-Villas, A., and Rambla de Argila, J. (2019). European Genome-Phenome Archive (EGA) - Granular Solutions for the Next 10 Years. In 2019 IEEE 32nd International Symposium on Computer-Based Medical Systems (CBMS) (ieeexplore.ieee.org), pp. 4–6.

17. Raisaro, J.L., Choi, G., Pradervand, S., Colsenet, R., Jacquemont, N., Rosat, N., Mooser, V., and Hubaux, J. (2018). Protecting Privacy and Security of Genomic Data in i2b2 with Homomorphic Encryption and Differential Privacy. IEEE/ACM Trans. Comput. Biol. Bioinform. *15*, 1413–1426.
