# Supplementary material for: CanDIG: Federated network across Canada for multi-omic and health data discovery and analysis
Source: Cell Genom. 2021 Nov 10;1(2):100033. doi: 10.1016/j.xgen.2021.100033 (PMC9903648; doi:10.1016/j.xgen.2021.100033)
Supplement: Document S3. Article plus supplemental information [file mmc3.pdf]

Cell Genomics

CellPress
OPEN ACCESS

Commentary

CanDIG: Federated network across Canada for multi-omic and health data discovery and analysis

L. Jonathan Dursi,[1,*] Zoltan Bozoky,[2] Richard de Borja,[3,13] Haoyuan Li,[4] David Bujold,[5] Adam Lipski,[4,6] Shaikh Farhan Rashid,[1] Amanjeev Sethi,[1] Neelam Memon,[4,24] Dashaylan Naidoo,[4,25] Felipe Coral-Sasso,[4,26] Matthew Wong,[7] P-O Quirion,[5] Zhibin Lu,[8] Samarth Agarwal,[9] Yuriy Pavlov,[1] Andrew Ponomarev,[4,10] Mia Husic,[11] Krista Pace,[1] Samantha Palmer,[1] Stephanie A. Grover,[12] Sevan Hakgor,[13] Lillian L. Siu,[13] David Malkin,[14] Carl Virtanen,[8] Trevor J. Pugh,[3,13,15] Pierre-Étienne Jacques,[16] Yann Joly,[17,18] Steven J.M. Jones,[4,19] Guillaume Bourque,[18,20,21] and Michael Brudno[1,22,23,*]

[1]DATA Team, University Health Network, Toronto, ON, M5G 2C4, Canada
[2]Providence Health Care, Vancouver, BC, V6Z 1Y6, Canada
[3]Ontario Institute of Cancer Research, Toronto, ON, M5G 0A3, Canada
[4]Canada's Michael Smith Genome Sciences Centre, BC Cancer Research Institute, Provincial Health Services Authority, Vancouver, BC, V5Z 4S6, Canada
[5]Canadian Centre for Computational Genomics, Montréal, QC, H3A 0G1, Canada
[6]Zymeworks, Vancouver, BC, V6H 3V9, Canada
[7]University of Waterloo, Waterloo, ON, N2L 3G1, Canada
[8]University Health Network, Toronto, ON, M5G 2C4, Canada
[9]University of Toronto, Toronto, ON, M5T 3A1, Canada
[10]GenX, Nottingham, NG1 1GF, UK
[11]Centre for Computational Medicine, The Hospital for Sick Children, Toronto, ON, M5G 1X8, Canada
[12]Genetics and Genome Biology Program, The Hospital for Sick Children, University of Toronto, Toronto, ON, M5G 1X8, Canada
[13]Princess Margaret Cancer Centre, University Health Network, Toronto, ON, M5G 2C1, Canada
[14]Division of Haematology/Oncology, The Hospital for Sick Children, Department of Pediatrics, University of Toronto, Toronto, ON, M5G 1X8, Canada
[15]Department of Medical Biophysics, University of Toronto, Toronto, ON, M5G 1L7, Canada
[16]Département de biologie, Université de Sherbrooke, Sherbrooke, QC, J1K 2R1, Canada
[17]Centre of Genomics and Policy, Department of Human Genetics, McGill University, Montreal, QC, H3A 0C7, Canada
[18]Department of Human Genetics, McGill University, Montreal, QC, H3A 0C7, Canada
[19]Department of Medical Genetics, University of British Columbia, BC, V6H 3N1, Canada
[20]Canadian Center for Computational Genomics (C3G), McGill University, Montreal, QC, H3A 0G1, Canada
[21]McGill Genome Center, Faculty of Medicine, McGill University, Montreal, QC, H3A 0G1, Canada
[22]Department of Computer Science, University of Toronto, Toronto, ON, M5T 3A1, Canada
[23]Vector Institute, Toronto, ON, M5G 1M1, Canada
[24]Present address: Vancouver, BC, Canada
[25]Present address: Molecular Genomics Laboratory, Providence Health & Services, Portland, OR, USA
[26]Present address: Florianopolis, 88037-400, Brazil
*Correspondence: jonathan.dursi@uhn.ca (L.J.D.), brudno@cs.toronto.edu (M.B.)
https://doi.org/10.1016/j.xgen.2021.100033

We present the Canadian Distributed Infrastructure for Genomics (CanDIG) platform, which enables federated querying and analysis of human genomics and linked biomedical data. CanDIG leverages the standards and frameworks of the Global Alliance for Genomics and Health (GA4GH) and currently hosts data for five pan-Canadian projects. We describe CanDIG's key design decisions and features as a guide for other federated data systems.

Canada is a confederation of provinces, each with its own health data privacy legislation, and data generated in each province must follow corresponding provincial laws. When we considered how to design a data sharing infrastructure for pan-Canadian human biomedical research projects, the diversity of regulations and legal frameworks across provinces meant there were very specific technical and privacy requirements, including (1) connecting distributed data under local control; (2) supporting data remaining on-premises; (3) simultaneously supporting multiple research in different domains, such as rare-disease and cancer research; (4) making use of existing compute, data, and authentication infrastructure as much as possible; (5) focusing first on enabling data discovery and querying, then analysis; and (6) a transparent open-source and standards-based approach for trust and interoperability.

While a common approach to data sharing is aggregation of large datasets in central repositories,[1] a federated approach was better suited to our framework across Canadian provinces.[2] Our requirements for transparent, open-source and standards-based approaches led us to adopting the international GA4GH technical and policy standards.[3] Implementing GA4GH standards in responsible data sharing (web resources), data security (web resources),

This is an open access article under the CC BY license (http://creativecommons.org/licenses/by/4.0/).

variant representation,[4] authentication,[5] and consents,[6] allowed our small team to quickly set up CanDIG, as well as to rapidly iterate on the platform and collaborate with groups internationally performing similar work and sharing lessons learned.

CanDIG is a Canadian national health research data platform, designed to support consented health research data discovery, querying, and analysis across centers and projects. CanDIG is the first multi-project human genomics and biomedical data federation in Canada, connecting the country's largest human sequencing centers (CGen; web resources). Deployed as a software stack at each site that joins the close governance of the federation, CanDIG has, to date, incorporated genomic and phenotypic data from five leading Canadian projects, including three projects spanning provincial boundaries: the Terry Fox Comprehensive Cancer Care Centre Consortium Network (TF4CN) and Terry Fox PRecision Oncology For Young peopLE (PROFYLE), the Canadian COVID-19 Genomics Network human sequencing project (CanCOGeN HostSeq), and two regional projects, POG (Personalized Onco-Genomics) and INSPIRE.[7] CanDIG participants include McGill University, The Hospital for Sick Children, University Health Network, Ontario Institute for Cancer Research, Canada's Michael Smith Genome Sciences Centre, Jewish General Hospital, and Université de Sherbrooke.

Here, we describe the choices we made building CanDIG—the data platform (the software stack and its operations) and the CanDIG Federation (the stakeholders, and the governance and policy between them that permits data access)—and compare them to other data federations for health research data. We first place CanDIG's platform in a three-dimensional landscape of data federations, considering range of queries, range of data types, and degree of decentralization, and compare it to well-known federated platform models such as the DataSHIELD,[8] Matchmaker Exchange,[9] Beacon network,[10] and the planned Federated EGA.[11] We then go into greater detail on the reason for our implementation choices and discuss technical details. Next, we describe the division of responsibilities and accountabilities in the federation, which is closely intertwined with the technical implementation.

In "CanDIG's implementation of GA4GH standards and technologies," we discuss the choice to adopt GA4GH standards, how those standards and collaborations allowed us to move faster and learn from other federations, and which standards we adopted immediately and what we have plans to adopt in the next version. We then discuss what a user has access to on the project dashboard and with the application programming interfaces (APIs) and conclude with future plans for developing and expanding CanDIG.

Federated data platform models

Federated data systems span a variety of arrangements.[2] Here, we refer to federation in terms of the connection of "horizontal partitions" of data, connecting geographically separated research cohorts where the data for various participants can be found at multiple sites. We do not consider linking multiple separate data sources or types for the same data subject—clinical data in one store, genomic data in a second store, crossing "vertical partitions." In our model this happens internally to a site, and we refer to those operations as performing data integration, rather than federation. We also distinguish between data that is merely distributed, falling upon a user to discover, query, and assemble results by themselves, and data within a federated platform, where the nodes coordinate and communicate among each other.

One of the key parameters for a data federation is the degree of decentralization (Figures 1A–1C), which describes how queries flow through the system and whether there are centralized or distributed identities.

Federated data platform models can be considered along two additional dimensions: (1) the level of access they provide to the data and (2) the diversity of datasets accessible via the federation. Figure 1D illustrated the flexibility of data federations to handle additional constraints, such as adding differential privacy to a query. Figures 1E and 1F categorize several well-known health data federations along these dimensions. For example, the Datashield[8] and the Local EGA projects[11] are "central access" or "hub and spokes" models (Figure 1B) with a central infrastructure and identities. Data access can also be approached in various ways, from having a few predefined queries, such as with the Beacon Network[10] and the Matchmaker Exchange (MME),[9] to running arbitrary analyses, such as with Datashield.[8]

Federated platforms must also be designed around the data types supported. Including a broader range of multi-omics, imaging, phenotypic and clinical data types is more valuable to researchers but increases complexity, may include more sensitive data, and makes federation governance a larger task.

CanDIG federated platform model

Given our requirements, and learning from successful health data federations described above, we chose to implement a fully distributed federated data platform. The CanDIG platform has no centralized infrastructure or data; coordination occurs through the collaboration of the sites and the governance, policy, and standards decisions at the national level. This avoids a number of governance issues—such as the location and jurisdictional policies of centralized infrastructure—and makes it easier to assure local data custodians of full control over their data. An additional requirement was to support a range of querying and processing methods on a wide variety of data types. Figures 1E and 1F illustrate CanDIG's position in our federated data platform design space.

A more detailed look at the implementation of these other federated data platforms (see Table S2) demonstrated that to be consistent with our decentralized approach and our requirement to make use of existing infrastructure wherever possible, authentication would rely on the identities and authentication mechanisms of the participating sites. Users would log in with their home sites credentials rather than with a centralized CanDIG identity. Authorization decisions would have to be made locally at each site, based on the trusted federation-peer user identity and the nature of the request. Our requirement to allow a number of query and analysis methods over a plethora of different data types necessitate rather fine-grained authorization—allowing a user to access counts of data without necessarily allowing access to individual records, for instance, or allowing access to somatic cancer mutations

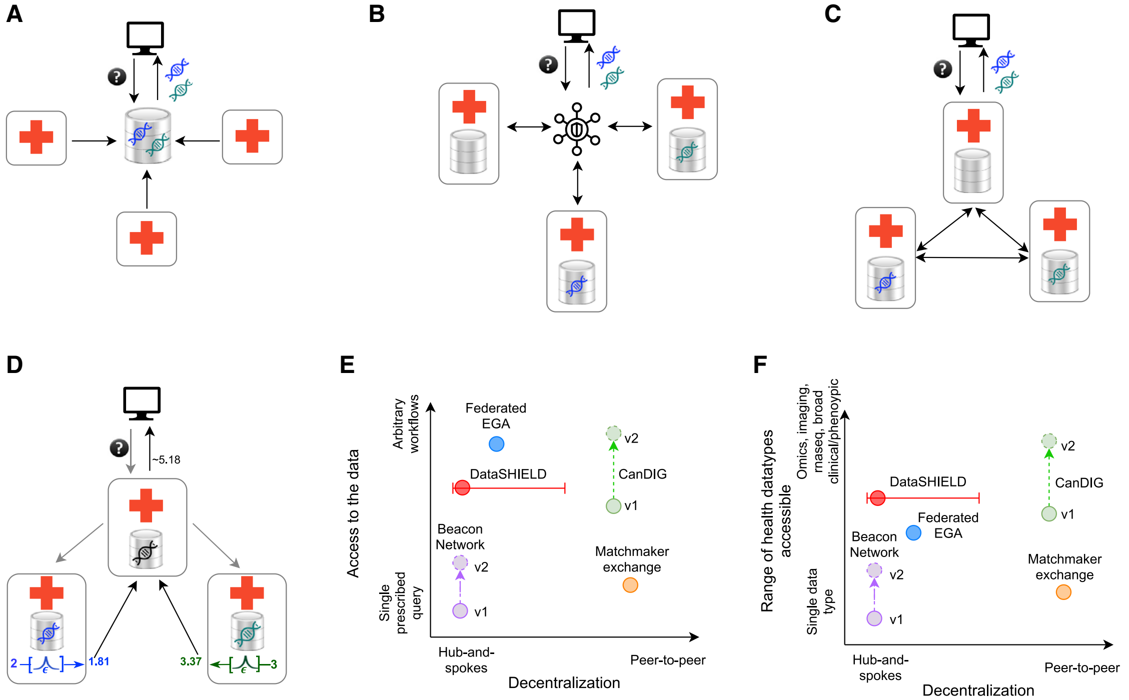

Figure 1. Degree of decentralization of a federated data platform

(A) A centralized (not federated) data repository where data is pulled manually or automatically into a central data store.

(B) A hub-and-spokes model of federation (a "central access model" as described in Thorogood et al.[2]), where there is significant central infrastructure that the peers are required to interact with.

(C) A decentralized, peer-to-peer network, where a user sends a request to a peer node—where relevant data may or may not be—and other peers are queried. Results (represented by DNA) are then returned to the user.

(D) Results can be locally processed at each site before being returned; in this case, perturbing results for the purposes of privacy enhanced analytics, in this case local differential privacy.[12] We can further categorize well-known health data federations along dimensions of decentralization (central-access to peer-to-peer).

(E) Level of access to data (pre-specified queries to arbitrary workflows).

(F) The range of different kinds of datasets supported by the various platforms.

but not germline variants. Details of our implementation can be found in supplementary notes section 1.

These requirements and design choices make for an approach quite different to other federated health data platforms. The requirement to support on-premises data meant moving to a centralized secure enclave in the cloud such as AnVIL[1] was not feasible. Unlike the Beacon network or DataShield, there is no central portal or infrastructure; unlike early versions of Local EGA, there is no central identity; unlike Matchmaker Exchange, all requests are made by an identified researcher user (and authorization decisions are based separately on that identity and local data entitlements).

CanDIG federation governance model

The CanDIG platform's design has iteratively co-evolved with the governance of the CanDIG Federation, which defines the roles, responsibilities, and accountabilities of all stakeholders.

In our multi-institution, multi-project federated data effort, key stakeholders include the national platform, the data-hosting sites, and the data programs. All play a role in governance and setting the direction of the project. The national platform is responsible for technical decisions (on software development, architecture, standards, and data modeling) necessary for implementing a data platform consistent with requirements and convening discussions and building consensus around those requirements. The participating sites are responsible for maintaining the CanDIG software stack at their institution, connecting it to local infrastructure (such as compute and identity management), following operations and security policy and contributing to the development effort. The data programs are the custodians for their local or distributed datasets, and own the relationships with the data subjects, are responsible for obtaining patient consents, data quality, and harmonization across their sites, working with the platform team to map to common data models, removal of direct identifiers, and communicating authorization decisions from their data access committees to the platform. Further details on roles and responsibilities are discussed in supplementary notes, section 3.

CanDIG platform adoption of GA4GH standards and technologies

Making use of existing and emerging GA4GH standards and frameworks[3] where applicable helped CanDIG start quickly on its federation by giving us relevant technical, policy, and data model standards we could work with immediately. GA4GH standards were extremely current, and actively maintained, and so facilitated

collaborations such as our involvement in the Africa-Canada-EU CINECA project for federated analysis of human cohort data (web resources) which is committed to following GA4GH standards.

We prioritized standard adoption by how well they fit into our platform and federation design. As one example, an early task was to flesh out the roles, responsibilities, and accountabilities described above in greater detail. We used standards and frameworks, including the Framework for Responsible Data Sharing (web resources) and Data Security Infrastructure Policy (web resources) as a ready and comprehensive list of responsibilities for health data handling that we could ensure were clearly assigned to one stakeholder or explicitly shared between two stakeholders.

On the technical side, the first version of CanDIG APIs are built on top of the code for the initial Genomics APIs (GA4GH Server; web resources) developed at the University of California Santa Cruz for GA4GH. These Genomics APIs have now been discontinued by the GA4GH in favor of other standards, but using this code base allowed us to start working immediately with data custodians to make data available, and to build our authentication, authorization, and query federation framework atop of an existing codebase. Our authentication and authorization approach is described in supplementary notes section 1, including the adoption of an API gateway. Use of the gateway as a common external interface to our components allowed us to begin adding additional services and APIs, and replacing others, while making use of the same authentication, authorization, and query federation. This made it easier to adopt new services and APIs. Current core functionality of a CanDIGv1 site includes adopted GA4GH standards such as the Data Use Ontology (DUO),[6] which we use to document consents required for individual datasets; RNAGet (web resources) and htsget,[13] which we have implemented ourselves as standalone services, and Beacon,[10] as well as preexisting standards that have been adopted by the GA4GH standards process such as CRAM/SAM/BAM, and VCF. In addition, DRS (web resources), and WES (web resources) are already being used internally at some sites.

The version currently in development, CanDIGv2 (web resources), includes those services as well as our implementations service-registry (web resources) to itemize the growing number of services available at a site, and Phenopackets (web resources) for structuring and returning phenotypic data for infectious disease or rare disease projects. In addition, Visa claims of the GA4GH Passport standard[5] are being used as a standard format to communicate data entitlements for a research user within a site. Finally, we are testing the use of GA4GH Variant Representation[4] as a common indexing mechanism for variants to solve the problem of allowing research users to perform variant queries in a number of different formats. Figure S1 shows how these tools and standards come together in the platform.

CanDIG use by pan-Canadian projects

CanDIG currently makes genomic and phenotypic data available to scientists across Canada and international collaborators as part of data sharing for five leading pan-Canadian projects, including the Terry Fox Comprehensive Cancer Care Centre Consortium Network (TF4CN) and Terry Fox PRecision Oncology For Young peopLE (PROFYLE[14]), as well as making human variant data from the Canadian COVID Genomics Network (CanCOGeN HostSeq; web resources) discoverable. It likewise makes data from provincial or single-site projects such as Personalized Onco-Genomics (Personalized Onco-Genomics; web resources) and the INSPIRE study[7] more accessible (current projects can be found listed on Table S1). These five projects, which include genomes and health data for nearly 2,000 study subjects, typically share their data via CanDIG so that users can discover subsets of relevant participant data (“data discovery”) and explore that subset interactively.

CanDIG supports both controlled access and registered access research users. Controlled access is explicitly granted by data access committees, and researchers with controlled access entitlements can see and query (via the dashboard or programmatically via queries; see next session) significant amounts to those datasets. We also have a growing number of registered access users[15] who have signed up and agreed to terms of service but have very limited querying ability, and only to those datasets (currently just CanCOGeN HostSeq) that have opted in to such access.

Data access through CanDIG: Dashboard and queries

CanDIG provides web-based dashboards, and programmatic querying via APIs, of the datasets. Users generally start with the dashboard.

Initial panes of the dashboard include simple overviews of the data in a dataset such as count of data subjects by geography, demography, and broad phenotypic categories, as well as indicating what molecular data types (variants, reads, RNA expression) are available. These overviews are useful initial introductions to a dataset, and can be the main requirement for a project manager keeping track of the progress of a project; relatively modest levels of authorization are needed to be able to access the data for these panes (Figures 2A–2C).

Researchers with higher levels of controlled access can have deep access to the data, allowing them to dig into individual cases. This too can be done via the dashboard, which allows viewing mutations by gene (Figure 2D), integrated IGV for viewing variants and their sequencing context (Figure 2E), and information about the analysis pipeline producing those results (Figure 2F).

We also enable programmatic access to data in CanDIG via APIs. In addition to the APIs discussed above, CanDIG has implemented an initial set of cross-service queries that allow querying for patients that have given clinical, variant, and expression data features, integrating the results from multiple APIs. Use cases include programmatic data discovery—identifying and querying relevant subsets of data based on a set of criteria—in a potentially automated way, as well as data analytics. As an example, we have demonstrated the ability to use these APIs for privacy enhancing machine learning (Figure 1C and supplementary notes section 4). We have trained a classifier on genomic and clinical data that uses the cross-service counts query with our initial implementation of local differential privacy,[12] a method of privacy enhancing analytical queries with provable limits on leaking of private information based on perturbing query results.

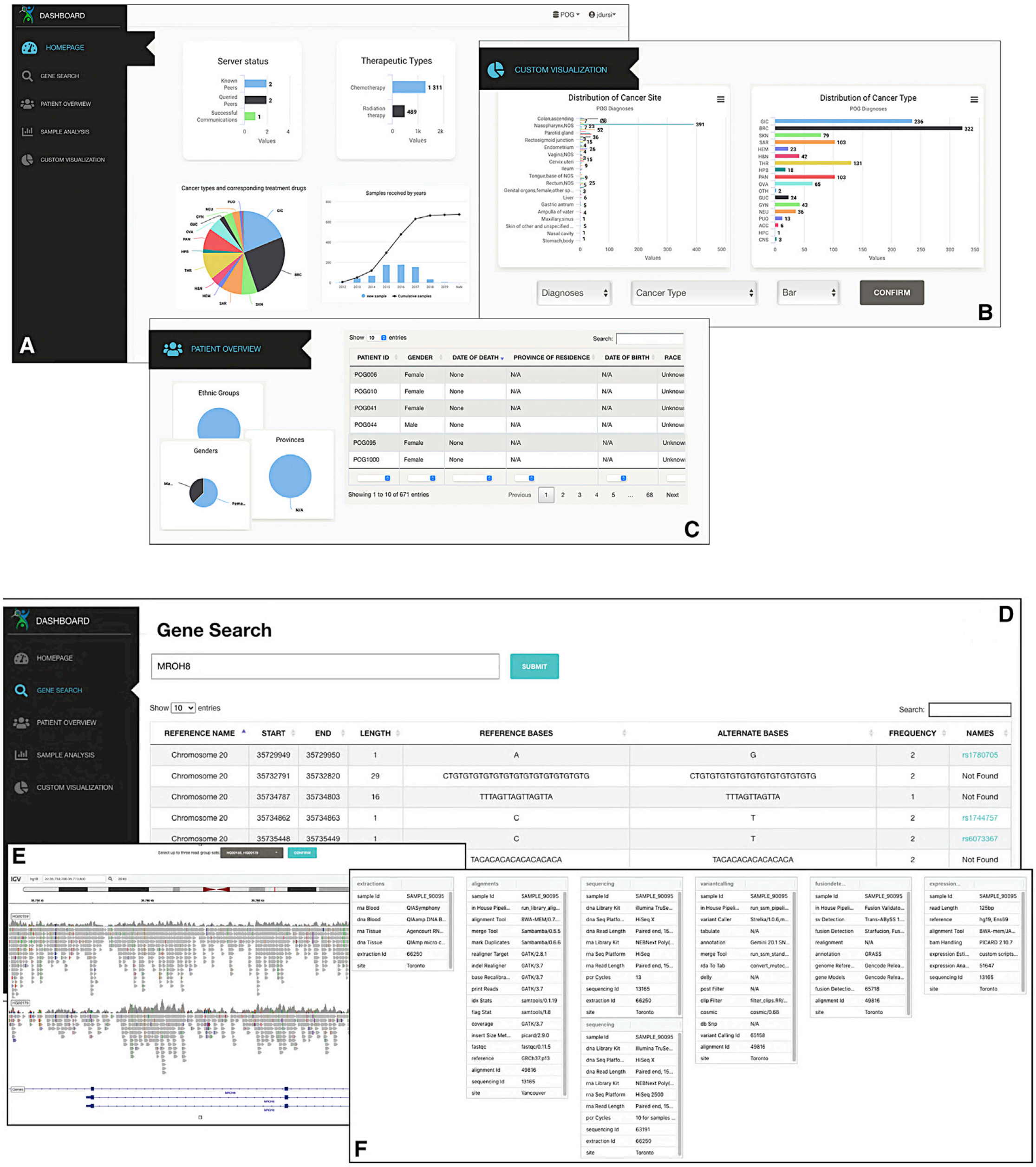

Figure 2. Using the CanDIG portal

CanDIG dashboard overview pages (for a research project manager, or someone examining a dataset for the first time), and more detailed pages (for authorized users). The website, and in particular the dashboard, is reactive and mobile-friendly, so that project status can be checked from anywhere.

(A–F) (A) The default simple summary statistics about participant data and cases available on the platform are shown. A project manager who wants to see breakdowns by other variables can use the custom visualization widget, illustrated in (B). Finally, the research project manager can quality check clinical data by a patient or check to see what associated genomic data is in the system through the patient's tab, illustrated in (C). A bioinformatician working with a particular sequencing project will want to explore genomic variants and read-level data, shown here with a federated mock dataset. A gene search page, shown in (D), allows querying variants in the dataset by gene name; from there the bioinformatician can view reads from the variant location in one or more participant's data in an embedded IGV.js tool, shown in (E). Information about the analysis pipeline used to generate the data from the sample collection to the variant calling tool settings can be shown through the sample analysis tab, shown in (F).

Discussion

As the first Canadian national human genomics and biomedical federated data platform, CanDIG provides researchers, clinicians, and their international collaborators the ability to access and analyze a Canadian pool of multi-omics and health data and enables simpler data sharing across projects both nationally and internationally. By connecting multiple national health data projects in Canada on a single platform, and implementing GA4GH standards,

CanDIG enables all to benefit from pan-Canadian datasets, as CanDIG and international GA4GH-standards compliant efforts can be queried jointly. Our involvement with GA4GH and an international community involved in data federation developments, also allowed us to build these systems faster, taking advantage of lessons, development efforts, and components developed in other systems.

The governance model of our fully distributed, multi-jurisdiction, multi-project platform makes explicit the roles and responsibilities of the platform, software development effort, sites, and data custodians. The clarity and separation of roles greatly eases participation in international federation efforts such as with the EU/Canada/Africa CINECA project. We believe that our governance model is portable to a number of other distributed health data projects that share data among trusted partners.

Based on what we have learned, we are continuing to develop CanDIG with a more extensible service-oriented architecture, which will allow more ready incorporation of more services, such as further support for workflows via GA4GH WES (web resources), additional authorization capabilities, data access committee portals, additional molecular data types, and more complex analytics. We also look to enable interoperability with clinical data by moving to a standard clinical data model (OMOP: web resources), querying of medical imaging metadata, and stronger ontology support. These additional capabilities are necessary to support the upcoming Digital Health and Discovery Platform (DHDP: web resources) project, with greater volumes and variety of data—but the fundamental distributed authentication, authorization, and federation approach underlying CanDIG will remain unchanged.

SUPPLEMENTAL INFORMATION

Supplemental information can be found online at https://doi.org/10.1016/j.xgen.2021.100033.

WEB RESOURCES

Canada's Genomic Enterprise (CGen), https://www.cgen.ca/

GA4GH Framework for Responsible Sharing of Genomic and Health-Related Data, https://www.ga4gh.org/genomic-data-toolkit/regulatory-ethics-toolkit/framework-for-responsible-sharing-of-genomic-and-health-related-data/

GA4GH Data Security Infrastructure Policy, https://github.com/ga4gh/data-security/blob/master/DSIP/DSIP_v4.0.md

Common Infrastructure for National Cohorts in Europe, Canada, and Africa (CINECA), https://www.cineca-project.eu/

GA4GH Server, https://github.com/ga4gh/ga4gh-server

RNAget implementation, https://github.com/CanDIG/rnaget_service

GA4GH Data Repository Service (DRS) Schemas, https://ga4gh.github.io/data-repository-service-schemas/

GA4GH Workflow Execution Service (WES) Schemas, https://ga4gh.github.io/workflow-execution-service-schemas/

GA4GH Service Registry API, https://github.com/ga4gh-discovery/ga4gh-service-registry

Phenopackets, http://phenopackets.org/

CanDIGv2 software stack, https://github.com/candig/candigv2

CanCOGeN HostSeq, https://www.genomecanada.ca/en/cancogen/cancogen-hostseq

Personalized Oncogenomics Program (POG), https://www.bcgsc.ca/personalized-oncogenomics-program

GenAP, Genetics & Genomics Analysis Platform, https://genap.ca/

ID3 Decision Tree Classifier used to interact with the CanDIG server, implemented to allow differential privacy to protect PHI, https://github.com/CanDIG/id3-variants-training

OMOP-CDM, https://www.ohdsi.org/data-standardization/the-common-data-model/

DHDP, https://www.dhdp.ca

ACKNOWLEDGMENTS

CanDIG development was funded by the Canada Foundation for Innovation Cyber infrastructure grant 34860, CANARIE Research Data Management contracts RDM-090 (CHORD) and RDM2-053 (ClinDIG), and the Canadian Institutes for Health Research as part of the Africa-Canada-EU Horizon2020 CINECA project (CIHR grant number #404896). P.E.J. is a research scholar from the Fonds de la recherche du Québec en santé (FRQS). G.B. is a Canada Research Chair in Computational Genomics and Medicine. M.B. is a CIFAR Canada AI Chair.

AUTHOR CONTRIBUTIONS

Conceptualization, M.B., G.B., S.J.M.J., L.J.D., and C.V.; Software, L.J.D., Z.B., R.d.B., H.L., A.L., S.F.R., A.S., N.M., D.N., F.C.-S., M.W., Y.P., and A.P.; Resources, Z.L., P.-O.Q., M.B., C.V., S.J.M.J., and G.B.; Writing - Original Draft, L.J.D., Z.B., R.d.B., H.L., M.B., M.H., and S.P.; Writing - Review & Editing, L.J.D., S.P., M.B., M.H., Z.B., R.d.B., H.L., D.B., A.L., S.F.R., A.S., N.M., D.N., F.C.-S., M.W., P.-O.Q., Z.L., S.A., Y.P., A.P., M.H., K.P., S.A.G., S.H., L.L.S., D.M., C.V., T.J.P., P.-É.J., Y.J., S.J.M.J., and G.B.; Funding Acquisition, M.B., G.B., S.J.M.J., Y.J., P.-É.J., T.J.P., C.V., L.L.S., and D.M.; Project Administration: K.P. and S.P.; Visualization: S.P. and H.L.; Supervision: M.B., G.B., S.J.M.J., Y.J., P.-É.J., T.J.P., C.V., and L.J.D.

DECLARATION OF INTERESTS

The authors declare no competing interests.

REFERENCES

1. Schatz, M.C., Philippakis, A.A., Afgan, E., Banks, E., Carey, V.J., Carroll, R.J., Culotti, A., Ellrott, K., Goecks, J., Grossman, R.L., et al. (2021). Inverting the model of genomics data sharing with the NHGRI Genomic Data Science Analysis, Visualization, and Informatics Lab-space (AnVIL). Cell Genomics *1*. https://doi.org/10.1101/2021.04.22.436044.
2. Thorogood, A., Rehm, H.L., Goodhand, P., Page, A.J.H., Joly, Y., Baudis, M., Rambla, J., Navarro, A., Nyronen, T.H., Linden, et al. (2021). International Federation of Genomic Medicine Databases Using GA4GH Standards. Cell Genomics *1*, 100032-1–100032-5.
3. Rehm, H.L., Page, A.J.H., Smith, L., Adams, J.B., Alterovitz, G., Babb, L.J., Barkley, M.P., Baudis, M., Beauvais, M.J.S., Beck, T., et al. (2021). GA4GH: international policies and standards for data sharing across genomic research and healthcare. Cell Genomics *1*, 100029-1–100029-33.
4. Wagner, A.H., Babb, L., Alterovitz, G., Baudis, M., Brush, M., Cameron, D.L., Cline, M., Griffith, M., Griffith, O.L., Hunt, S.E., et al. (2021). The GA4GH Variation Representation Specification: A Computational Framework for variation representation and Federated Identification. Cell Genomics *1*, 100027-1–100027-11.
5. Voisin, C., Linden, M., Dyke, S.O.M., Bowers, S.R., Reinold, K., Lawson, J., Li, S., Wang, V.O., Barkley, M.P., Bernick, D., et al. (2021). GA4GH Passport standard for digital identity and access permissions. Cell Genomics *1*, 100030-1–100030-12.
6. Lawson, J., Cabili, M.N., Kerry, G., Boughtwood, T., Thorogood, A., Alper, P., Bowers, S.R., Boyles, R.R., Brookes, A.J., Brush, M., et al. (2021). The Data Use Ontology to streamline responsible access to human biomedical datasets. Cell Genomics *1*, 100028-1–100028-9.
7. Cindy Yang, S.Y., Lien, S.C., Wang, B.X., Clouthier, D.L., Hanna, Y., Cirlan, I., Zhu, K., Bruce, J.P., El Ghamrasni, S., Iafolla, M.A.J., et al. (2021). Pan-cancer analysis of longitudinal metastatic tumors reveals genomic alterations and immune landscape dynamics associated with pembrolizumab sensitivity. Nat. Commun. *12*, 5137.
8. Wilson, R.C., Butters, O.W., Avraam, D., Baker, J., Tedds, J.A., Turner, A., Murtagh, M., and Burton, P.R. (2017). DataSHIELD–new directions and dimensions. Data Sci. J. *16*, 1–21.

CellPress
OPEN ACCESS

9. Buske, O.J., Schiettecatte, F., Hutton, B., Dumitriu, S., Misyura, A., Huang, L., Hartley, T., Girdea, M., Sobreira, N., Mungall, C., and Brudno, M. (2015). The Matchmaker Exchange API: automating patient matching through the exchange of structured phenotypic and genotypic profiles. Hum. Mutat. *36*, 922–927.

10. Fiume, M., Cupak, M., Keenan, S., Rambla, J., de la Torre, S., Dyke, S.O.M., Brookes, A.J., Carey, K., Lloyd, D., Goodhand, P., et al. (2019). Federated discovery and sharing of genomic data using Beacons. Nat. Biotechnol. *37*, 220–224.

11. Fernández-Orth, D., Lloret-Villas, A., and Rambla de Argila, J. (2019). European Genome-Phenome Archive (EGA) - Granular Solutions for the Next 10 Years. In 2019 IEEE 32nd International Symposium on Computer-Based Medical Systems (CBMS), pp. 4–6, ieeexplore.ieee.org.

12. Duchi, J.C., Jordan, M.I., and Wainwright, M.J. (2013). Local Privacy and Statistical Minimax Rates. In 2013 IEEE 54th Annual Symposium on Foundations of Computer Science, pp. 429–438.

13. Kelleher, J., Lin, M., Albach, C.H., Birney, E., Davies, R., Gourtovaia, M., Glazer, D., Gonzalez, C.Y., Jackson, D.K., Kemp, A., et al.; GA4GH Streaming Task Team (2019). htsget: a protocol for securely streaming genomic data. Bioinformatics *35*, 119–121.

14. Grover, S.A., Berman, J.N., Chan, J.A., Deyell, R.J., Eisenstat, D.D., Fernandez, C.V., Grundy, P.E., Hawkins, C., Irwin, M.S., Jabado, N., et al. (2020). Terry Fox PRecision Oncology For Young peopLE (PROFYLE): A Canadian precision medicine program for children, adolescents and young adults with hard-to-treat cancer. Cancer Res. *80*, 5413.

15. Dyke, S.O.M., Kirby, E., Shabani, M., Thorogood, A., Kato, K., and Knoppers, B.M. (2016). Registered access: a 'Triple-A' approach. Eur. J. Hum. Genet. *24*, 1676–1680.

Cell Genomics, Volume *1*

Supplemental information

CanDIG: Federated network across Canada for multi-omic and health data discovery and analysis

L. Jonathan Dursi, Zoltan Bozoky, Richard de Borja, Haoyuan Li, David Bujold, Adam Lipski, Shaikh Farhan Rashid, Amanjeev Sethi, Neelam Memon, Dashaylan Naidoo, Felipe Coral-Sasso, Matthew Wong, P-O Quirion, Zhibin Lu, Samarth Agarwal, Yuriy Pavlov, Andrew Ponomarev, Mia Husic, Krista Pace, Samantha Palmer, Stephanie A. Grover, Sevan Hakgor, Lillian L. Siu, David Malkin, Carl Virtanen, Trevor J. Pugh, Pierre-Étienne Jacques, Yann Joly, Steven J.M. Jones, Guillaume Bourque, and Michael Brudno

Supplemental Information for CanDIG: Federated Network across Canada for multi-omic and health data discovery and analysis

Supplemental Notes

Section 1: CanDIG Implementation Approach
Section 2: A Granular Model of Data Federations
Section 3: Roles and Responsibilities in the CanDIG model
Section 4: Local Differential Privacy Proof of Concept

Supplemental Figures

Figure S1: Current and Coming Use of GA4GH standards in the CanDIG Platform
Figure S2: Division of Roles and Responsibilities to CanDIG Federation Stakeholders
Figure S3: Proof of Concept Implementation of Local Differential Privacy

Supplemental Tables

Table S1: CanDIG Datasets
Table S2: Six-Dimension Approach to Designing Data Federations
Table S3: Roles and Responsibilities of CanDIG Partners

Supplemental Notes

Section 1: CanDIG Implementation Approach

The CanDIG effort decided on several principles before building anything.

CanDIG:

- Is **fully distributed**: All data, and all infrastructure, is completely distributed; no shared or centralized services. All coordination is done at the level of policy, protocol, or software development.
- **Gives the sites full local control** of authorization to data: Consistent with common governance and policies, local data custodians have complete control over access to their data, and auditability/observability into data access and use. We do not support large-scale data transfers and downloads as they sacrifice later auditability.
- Is **API-first**: Since we are building a platform whose success hinges on the interaction between users and multiple sites, new development will rely on API-first design, with APIs developed and documented, and services and clients built on this. This ensures documentation of the APIs, interoperability between clients, and alignment with GA4GH efforts.
- **Supports private consented research data**: Our mission is to connect privacy-sensitive, although not directly identifying, human health data. We will rely on modern authentication and authorization technologies to that end, and follow GA4GH Security Working Group's best practices. Since CanDIG is also fully distributed there is no central infrastructure to maintain or secure. We don't intend to support non-private data (e.g., from model organisms) or unconsented data from routine clinical care.
- Is **Open Source, Standards-Based, and Interoperable**: CanDIG builds on existing standards, on matters both technical and genomic (via its role as a driver project for the GA4GH effort). We enthusiastically adopt software written elsewhere. This approach allows interoperability as wide as possible while focusing our efforts only where it addresses our particular needs.

CanDIG-server (web resources) is a core part of the v1 platform, implementing many of the APIs it supports (and all that are mandatory for a v1 node). In the CanDIG server, many API endpoints are built on the retired GA4GH reference implementation (GA4GH Server: web resources) – indeed, roughly half of the code in the CanDIG-server comes from the reference implementation – and thus inherits its style, and how they were implemented. HTTP RPC-style methods are used for data retrieval, where the POST endpoints have a pre-defined request and response structure in protobuf, whose JSON form is accepted. We've built on that to allow binary protobuf messages between sites where the size and serializability of the response is an issue. In early work, we added 23 sets of newly-added clinical and pipeline metadata endpoints, each permitting both a simple request and a complex search with filtering. We support a number of operators in the filter objects, including lt, le, eq, ne, ge, gt, contains, in, etc. To ensure the ease of use of our APIs, we provide a Swagger API documentation, whose definition is widely used and can be visualized via a number of tools. We also provide a number of sample queries

that cover most of the common use-cases. By implementing these searches consistently across services we enabled complex cross-datatype queries; future work will investigate the new standard GA4GH Data Connect for such searches. In addition, we added a clinical and phenotypic data model informed by rare-disease work and a late pre-mCODE standard for cancer studies[1].

Having an initial set of APIs over genomic and relevant clinical data allowed us to bootstrap our federation and distributed authentication and authorization infrastructure atop. In our decentralized federation, there is no central “CanDIG” identity; each user has a home site, typically the institution at which they work, where they can log in using their local credentials and can view the dashboard there. This propagates the queries necessary to drive the dashboard across to all federation partners. Currently, a simple, single-step fan-out is sufficient. Each site in the federation recognizes the identity of the other sites and makes authorization decisions regarding access to the data it hosts. The granularity of that authorization allows for multiple projects to be supported without exposing data from one project to researchers of another unless so authorized. That information is then presented to the user via their home site. Because the user is necessarily authorized to see the data from the response, and the home site is effectively the user’s work computer, the requests can be safely aggregated at the home sites. Other topologies and communication strategies have been tested and will be used as the number of our participating sites grows.

Key to our authentication is open-source software components: Keycloak and Tyk, one of which exists at each site. Managing the Keycloak instance is a shared responsibility between the CanDIG developers and the local host site IT team; it allows users to log in using research institution or hospital credentials, allowing trusted federation partners to handle identity management, while providing OpenID Connect identity tokens. Keycloak is connected to each site’s internal authentication service and does not store any credentials itself. Keycloak has out-of-the-box support for active directory, LDAP, and Kerberos for internal network user registries. We do not federate identities; we recognize as part of the federation agreement the distributed identities supplied by the federation partners. Relying on standard tools has made it easy to extend our approach to authentication - as part of the CINECA project, we have shown that we can conditionally accept ELIXIR OIDC tokens (CINECA: web resources) as an identity token. We had to fix a bug in Keycloak (web resources) to do it, but by doing so we contribute to the entire ecosystem of users interested in using GA4GH passport tokens.

Services rely on the user's OIDC Identity Token associated with each request - after a final validation – to authorize data requests. In the CanDIG model, authorization is informed by platform-level information, but authorization decision-making is always strictly local. Currently, authorization information is maintained in a local text-based database; users with different project roles or areas of specialty are granted different "access levels" to a dataset by the study's DAC, with each record of clinical and phenotypic data belonging to a dataset and each property within the record having a default access level. However, the required access levels of each item - each property of any particular row - can be increased, allowing, for instance, data custodians responsible for data from marginalized populations to require additional levels of

authorization to access the study data, reinforcing privacy protections for individuals particularly vulnerable to medical and other discrimination.

At each site, the open-source API gateway Tyk serves as a "single sign-on" for any services behind it; it validates the OIDC tokens from any of the federation Keycloak instances, and once the request has a recognized identity token it allows rewriting or rerouting of requests enabling us to maintain a static external set of APIs while changing internals. Tyk also has essential features such as session handling, rate limiting and logging of incoming requests. We implemented middleware to also handle the "OAuth2 dance", to perform the 3-way authentication handshake involving the client and Keycloak, so that Tyk serves as the "Relying Party" for OIDC/OAuth2.

With the authorization in place, complex queries that researchers wish to perform programmatically or that are awkward to use a web interface for are available through increasingly rich APIs connecting the individual services and APIs supported by the platform. These components were unified through a single /search endpoint returning the information, or /count which returns counting aggregations, implemented in the candig-server codebase.

Once the distributed identity, local authorization, APIs, and API gateway were in place, it was straightforward to begin refactoring and adding additional capabilities. The federation component, originally built into candig-server, has been pulled out into its own service with extended functionality; this allows us to integrate new services, with one site supporting RNAGet and two supporting htsget, allowing us to both provide needed functionality early and test-driving new services and methods for integration into next versions of the software implementation.

With the federation approach now solid and battle-tested, the bootstrapping and learning approach we've taken with the development of the federation and platform are now able to more rapidly iterate. We now are actively working on version two (CanDIG V2: web resources) of the software stack, with technical write-ups underway, and have plans for version three (including a very different approach to clinical data modelling (OMOP Service: web resources), using the well-known OHDSI OMOP Common Data Model (web resources)), but crucially the basic federation approach and overall architecture will differ very little. Our implementation of authorization will grow more sophisticated in some ways (web resources), with a policy engine (OPA: web resources) simplifying the coordination of authorization across multiple services and addition of DAC portals using ELIXIR's REMS (web resources) tool; and less complicated in others – while the per-field granularity of v1's authorization was desired by some data custodians, in practice it was rarely used and will be handled more simply. But crucially the basic approach of local authorization informed by federation-level information remains the same, and only the box names on an architecture diagram change.

What we've learned and are iterating on also includes operations. We've gained experience working with local site managers on shared tools that cross the CanDIG/site service boundaries with Keycloak. Work underway for the next version which will support larger scale and

automation, we have defined new boundaries. MinIO and GA4GH Data Registry Service (DRS), already implemented at one site, mark the standard APIs for storage which the CanDIG stack can leverage but allow sites to use a variety of back-end storage systems. Similarly, the GA4GH Workflow Execution Service (WES) (web resources) and the Common Workflow Language (CWL)[2] implemented at two but not yet exposed to the user allow us to decouple the definition and invocation of computational pipelines from how they are run on back-end computational infrastructure.

To support standards and interoperability, we build wherever possible on existing technical and genomic standards. As mentioned earlier, we use OIDC for authentication, and OpenAPI for defining APIs; we rely on standard genomics formats like CRAM and VCF; we use and help shape GA4GH APIs such as Htsget, Beacon, and RNAget, GA4GH ontologies such as DUO for consented use for data. Upcoming work will use standards in exposing services such as CWL, GA4GH WES, and DRS. We have established the success of this approach in promoting interoperability by demonstrating initial two-way authentication with the ELIXIR Authentication and Authorization Infrastructure[3].

Section 2: A Granular Model of Data Federations

We have employed a six-dimension approach to describing the design of data federations, using the distinctions outlined below (Supplementary Table 2). Foundationally, a governance model which includes how federation peers join and leave, and the trust model required between them; how authentication of federation users work; the granularity of authorization; what queries are enabled; how queries flow through the federation; and how intermediate data is combined to be presented to the researcher.

In MME, researcher identities are only meaningful at each node, and the nodes themselves make requests of each other.

The depth of access to data often reflects deeper cooperation and shared governance between the data sites. Federations can be quite open, allowing new peers readily, such as the Beacon Network, or closed, allowing deeper access to the data or access to sensitive data but requiring formal agreements to be signed to join (such as Datashield, with deep access to data, or MME with access to deeply phenotyped childhood rare disease data).

We have a strong trust model between our participating institutions. They are all teaching hospitals and research institutions with long histories of collaboration and experience signing and honouring agreements with each other. A primary use case for CanDIG is to support national projects that the sites are collaborating on. While we do not expose raw data between sites, this strong level of trust gives us greater flexibility in the release of and combination of intermediate results in analyses.

Platform authentication is performed at the institution level – each institution provides a strong identity for its CanDIG users, and each user must have a CanDIG institution vouching for them. All requests in the platform are tied to a single user, ensuring enforceable accountability at the level of the researcher and the institutions. For authentication, we use the web—standard OpenID Connect (OIDC)[4] technology.

For controlled access data, access authorization decisions are made locally by the data sites. In our case, these sites bear ultimate responsibility for incorrectly authorized data release. However, many of the data sets stored within CanDIG are part of larger national projects which have data access committee lists maintained by one of the sites. Thus, the local authorization takes as input external data.

Query flow through our system is entirely peer-to-peer. Since every user "belongs" to an authenticating site, their queries can flow to that site, whence they propagate outwards through the closed federation to be received at peer sites. All requests through the system are associated with the single authenticated researcher who made the request; this is simplified by our adoption of OIDC authentication. While we have experimented with peer-to-peer cycle topologies to enable certain types of privacy-enhancing aggregations, for the time being, we use a simple fan-out topology.

For result aggregation, we make use of the fact that each user is strongly associated with a particular site, and so results the user is entitled to access can be safely aggregated on that site. We have also experimented with the use of homomorphic encryption to allow for global approaches to differentially private aggregations. This requires lower amounts of total noise (and thus higher result utility) to maintain privacy than our current local (or regional) differential privacy approach but is not implemented currently.

Finally, CanDIGv1 does not automate any form of platform-wide observability or auditability of access patterns across the closed federation as a whole, given the select number of sites, the closely-knit team, and the modest query rate, this has not been necessary. Approaches are being proposed for v2.

Section 3: Roles and Responsibilities in the CanDIG model

With a "multi-tenant" platform supporting multiple research projects, and federation separating the roles of sites and the platform as a whole, the CanDIG project has had to be very explicit about the roles and responsibilities of each partner in its role (Supplementary Figure 2). Below is a more detailed listing of the roles and responsibilities outlined above (see Supplementary Table 3).

Section 4: Local Differential Privacy Proof of Concept

The CanDIG platform provides authorized researchers access to complex queries and analyses of distributed datasets, jointly across various supported data types. This is enabled through very fine-grained authorization. Staff and researchers with different project roles or areas of specialty are granted different "access levels" to a dataset by the study's DAC, with each record of clinical and phenotypic data belonging to a dataset and each property within the record having a default access level. However, the required access levels of each item - each property of any particular row - can be increased, allowing, for instance, data custodians responsible for data from particularly marginalized populations to require additional levels of authorization to access the study data.

With the authorization in place, complex queries that researchers wish to perform programmatically or that are awkward to use a web interface for are available through increasingly rich APIs connecting the individual services and APIs supported by the platform. These components were unified through a single /search endpoint returning the information, or /count which returns counting aggregations. These APIs supported by the platform permit researchers to programmatically perform complex queries that are not supported by the user interface. Such queries can serve both discovery and simple analyses use cases.

For custodians who are considering making data available for discovery queries to a broader range of researchers while maintaining participant privacy, CanDIG has demonstrated support for making privacy enhanced analytics queries available as counts with local differential privacy [5] as an initial proof of concept. Laplace or exponential noise can be added to counts at each site before being summed and presented to the user (Figure 1A). This can be used for data discovery, but is also enough to support complex analyses. For instance, it is possible to train ID3 machine-learning classifiers [6] to predict ancestral data from a modest number of informative SNPs in the 1000 genomes data using the existing counts functionality (ID3 Decision Tree Classifier implementation can be found here: https://github.com/CanDIG/id3-variants-training). To illustrate this we perform queries on the client side that gather counts of a small set of known-informative SNPs - a subset of 17 from a known panel of 55 [7], grouped by demographic information (the 1000 genomes ancestry), and train a decision tree classifier based on the "splits" of ancestry by presence or absence of the SNPs. ID3 decision trees work particularly well with differentially private aggregations due to their use of counts (Figure S3).

It is also possible to use the APIs to perform more traditional population genetics visualizations such as identifying SNP counts versus ancestry. Our experience with these workflows is informing the continuing development of cross-service APIs for analytics, pushing the analyses to the server rather than the client.

Supplemental Figures

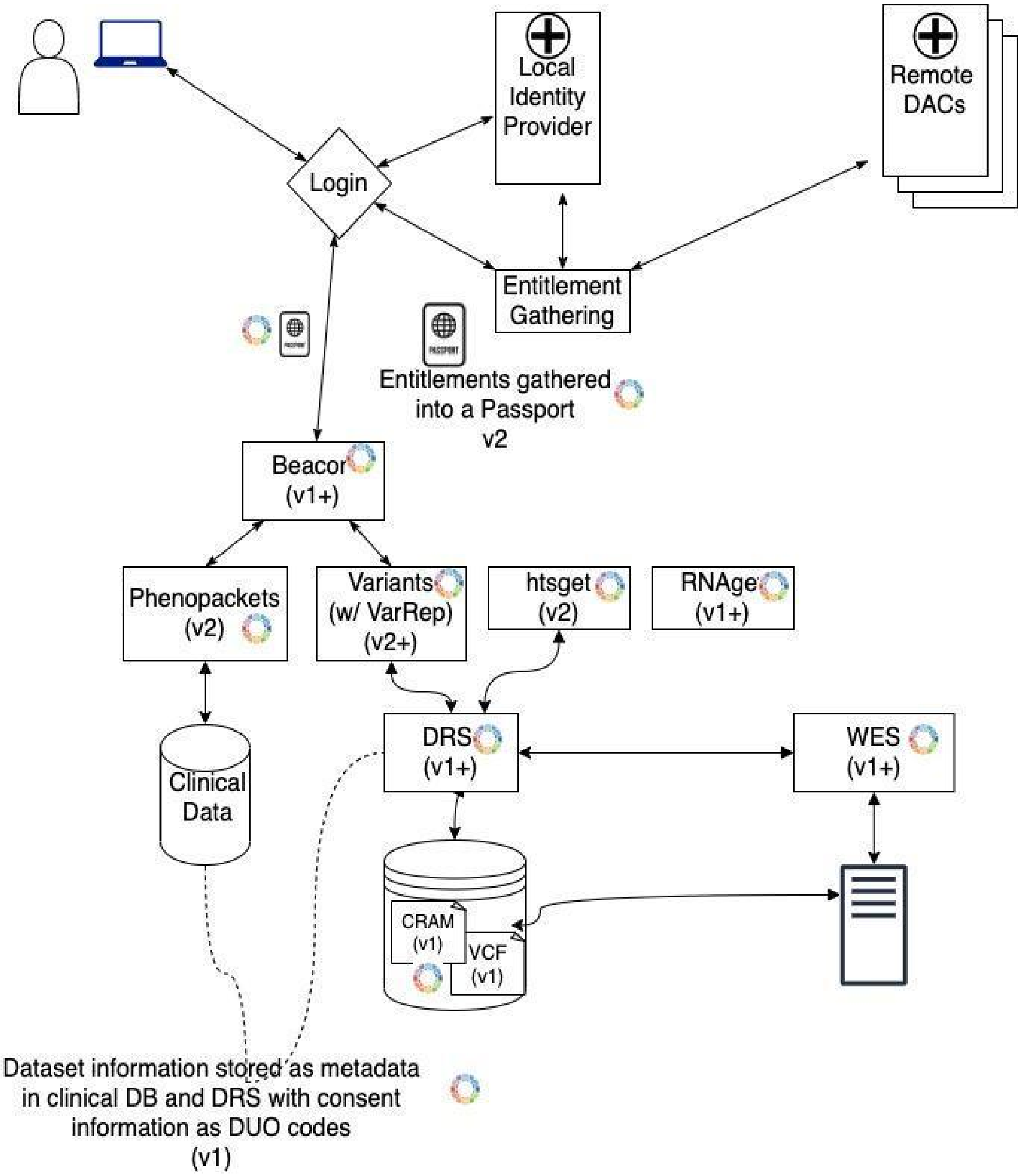

Figure S1: Current and Coming Use of GA4GH standards in the CanDIG Platform, related to CanDIG Platform Adoption of GA4GH Standards and Technologies. A schematic diagram illustrating how GA4GH technical standards are being used in the evolving CanDIG platform as we move towards deploying CanDIGv2. Illustrated are representations of services and standards being deployed within an individual CanDIG site, and how they build upon each other - use of standard Phenopackets eases the lightning of Beacons, a standard Variant Representation will avoid ambiguity of variants, the Data Repository Service powers Workflow Execution Service as well as other file-based services such as htsget, and internally entitlements are propagated using a representation based on Passport visas. Consent metadata uses ontologies like DUO. Not shown are services following similar patterns as others, such as RNAget or refget, and that each service identifies itself using Service-Info. Also not shown are the use of policy standards such as the Framework for Responsible Data Sharing, Breach Reporting, or Privacy and Security Policy. Tools that are a mandatory part of CanDIGv1 are noted with (v1); those that are deployed but not mandatory in v1 are noted with (v1+); those that additionally are required as part of CanDIGv2 are noted with (v2); those that are in development for future versions of v2 are noted with (v2+).

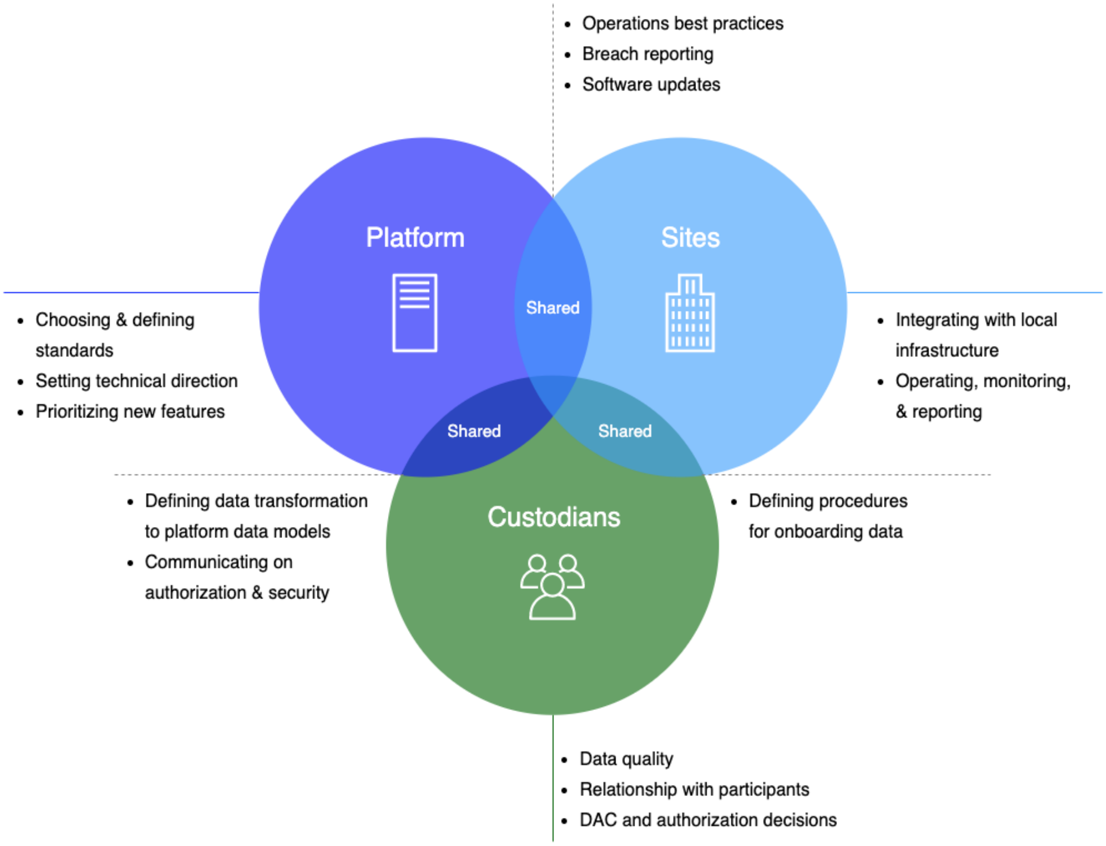

Figure S2: Division of Roles and Responsibilities to CanDIG Federation Stakeholders, related to CanDIG Federation Governance Model. A high-level Venn diagram of roles and responsibilities of CanDIG Platform Partners - the data custodians (such as research projects), the platform effort, and the individual sites. A more detailed list of the responsibilities of each partner is shown in Supplementary Table 3.

A

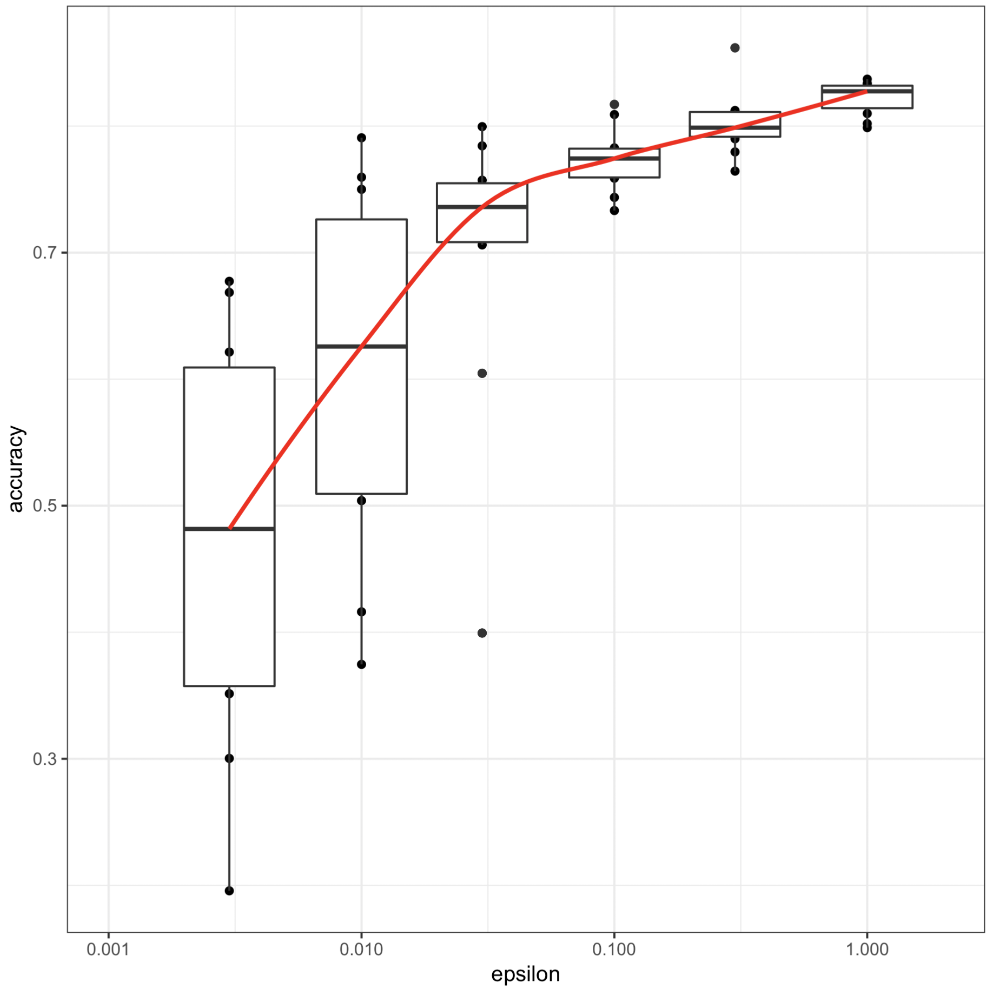

B

```
ID3 (label, dataset, cur_ethnicity_counts, has_variants, lacks_variants, remaining_variants, dataset)
  node ← create_node(label=label, left=∅, right=∅)

  if |remaining_variants| = 0 || argmax cur_ethnicity_counts = sum(cur_ethnicity_counts)
    node.label ← argmax cur_ethnicity_counts
    return node
  best_ig, best_v, best_ec_left, best_ec_right ← -∞, ∅, ∅, ∅
  for v in remaning_variants:
    CanDIG API call: counts_left ← count(dataset, has_variant=v ∪ has_variants, lacks_variants, by=ethnicity)
    counts_right ← clip(cur_ethnicity_counts - counts_left)
    information_gain ← gain(counts_left, counts_right)
    if information_gain > best_ig
      best_ig, best_v, best_ec_left, best_ec_right ← information_gain, v, counts_left, counts_right

  node.left ← ID3(+best_v, best_ec_left, has_variants ∪ v, lacks_variants, remaining_variants - v)
  node.right ← ID3(-best_v, best_ec_right, has_variants, lacks_variants ∪ v, remaining_variants - v)
  return node

tree ← ID3(label=root, has_variants=∅, lacks_variants=∅, remaining_variants=variants, training_dataset)
```

Figure S3: Proof of Concept Implementation of Local Differential Privacy, related to Figure 1D. Consistent with our approach of ensuring local control over authorization decisions, for queries or datasets where differential privacy is required we demonstrate a proof of concept implementation of the privacy enhancing analysis technique of local differential privacy. In local differential privacy, each data site determines its privacy parameter ϵ and perturbs the results with a corresponding degree of Lapacian or exponential noise. Lower ϵ means lower privacy loss, requiring more noise and lower resulting accuracy. This contrasts with global differential privacy, where a central site adds a global level of noise after getting unperturbed data from the sites. **A** Demonstrates a proof of concept, training of an ID3 tree based on 17 known ancestry informative SNPs on five of the 1000 genomes superancestries, with per-query laplacian noise (determined by the query sensitivity and ϵ) added at each query, trained on 250 randomly selected training genomes and tested against held out samples. With decreasing levels of the privacy parameter ϵ, privacy increases and utility is diminished; accuracy varies more widely run to run with larger amounts of noise (smaller values of ϵ). Future implementations will make use of robust production-quality differential privacy implementations such as those of OpenDP (https://opendp.org/). **B** Illustrates the classification algorithm, an ID3 tree classifier, implemented on the client side.

Supplemental Tables

Project	Status	Number of Participants with data	Participating Sites	Genomic and other Data Types
Precision Oncogenomics (POG)[8]	Current	570	BC Cancer Canada's Michael Smith Genome Sciences Centre (BCGSC)	WGS - tumour and normal
INSPIRE [9–11]	Current	106	University Health Network (UHN)	WES - tumour and normal; RNA-Seq
TF4CN/COMPARISON	Current	12	UHN, BCGSC	WGS - tumour and normal; RNA-Seq
CanCOGen HostSeq (web resources) - COVID-19 Host sequencing data, initial project for CanDIGv2	Current	983 currently, rising to 10,000	BCGSC, with McGill to join shortly	WGS
PRecision Oncology for Young peopLE (PROFYLE)[12]	In progress	338	McGill, SickKids, BCGSC	WGS - tumour and normal; RNA expression
Digital Health and Discovery Platform (2021-2025)	Upcoming	Target: 15,000 over 4 years	Initially: McGill, Centre hospitalier de l'Universite de Montreal (CHUM), UHN, BCGSC	WGS - tumour and normal, imaging, extensive clinical phenotype (mCODE)

Table S1: CanDIG Datasets, related to CanDIG Use By Pan-Canadian Projects
Current and forthcoming projects supported by CanDIG, including available patient numbers and data types. CanDIG's federation and distributed authentication and authorization will be part of the DHDP (web resources), a national platform initially supporting the government of Canada and Terry Fox Research Institute-supported Marathon of Hope Cancer Centre Network (web resources), a 15,000-patient cohort-of-cohorts of consented cancer data.

Model	Governance & Peer Trust Model	AuthN	AuthZ granularity	Queries	Query flow	Results gathering
Beacon Network [13]	Open	Partial recognition of external identities	Multi-dataset; open, registered access, controlled access	Single-query	Hub-spoke	Aggregation to hub
MME [14]	Closed	Site-to-site	Single-dataset	Single-query	Pairwise	Pairwise aggregation
DataShield[15]	Somewhat closed; peers trust central portal	Central identity	Fine-grained	Many (~140) primitives	Hub-spoke	Summary statistics only
Local EGA[16]	Closed	Central identity	Multi-dataset	File access	Hub-spoke	Aggregation to requestor
SPHN [17]	Closed, low-trust	Recognition of node identities	Fine-grained, multi-dataset	Multiple APIs	Peer-to-peer	Secure multi-party computation, homomorphic encryption
CanDIG	Closed, high-trust	Recognition of peer identities	Fine-grained, multi-dataset	Multiple APIs	Peer-to-peer	Aggregation to requesting site; optional differential privacy

Supplementary Table 2: Six-Dimension Approach to Designing Data Federations, related to CanDIG Federated Platform Model
A comparison of different federation models along six dimensions - governance/peer trust, authentication, authorization granularity, query range, query flow, data gathering.

CanDIG Platform	Data Custodians	CanDIG Sites
The federation of CanDIG sites, and the coordination thereof. A national steering committee of PIs, advisors, and technical leadership.<br><br>Responsible for:<br><br>● Coordinating software development (which takes place at the sites)<br>● Policy setting, including adopting GA4GH-recommendations for best practices<br>● Coordinating operating best practices, developed at the sites<br>● Road-mapping based on user (via custodians) and operational (via site) input<br>● Standards adoption and development<br>● International interoperability<br>● Working with external collaborators<br>● Onboarding new data custodians/data projects<br>● Promoting the harmonization of data access conditions (authorizations) between local custodians	May be national (with data hosted at multiple sites) or local (with data hosted at one).<br><br>Responsible for:<br><br>● Interacting with participants (consent, withdrawal)<br>● De-identification<br>● Data quality<br>● Defining authorizations, authorized users, such as through a data access committee (DAC)<br>● Defining additional requirements and feature requests	Located at a health research institution; works closely with both local data custodians and the platform effort.<br><br>Responsible for:<br><br>● Integration of the stack with local identity management, compute/storage infrastructure<br>● Providing authentication and federated query handling for local users<br>● Operating best practices, backups, monitoring<br>● Sharing knowledge and best practices<br>● Keeping the software up to date<br>● Ingesting data from local custodians<br>● Maintaining peering with federation sites<br>● Reporting<br>● Enforcing up to date authorizations<br>● Incident response and breach reporting

Table S3: Roles and Responsibilities of CanDIG Partners, related to CanDIG Federation Governance Model.

Supplementary Material Web Resources

CanDIG Server, https://github.com/candig/candig-server

GA4GH Server, https://github.com/ga4gh/ga4gh-server

CINECA, Integration of new cohort infrastructures to the ELIXIR AAI,

https://www.cineca-project.eu/blog-all/integration-of-new-cohort-infrastructures-to-the-elixir-aai

Keycloak, Pull Request, https://github.com/keycloak/keycloak/pull/7214

CanDIG V2 https://github.com/candig/candigv2

OMOP Service, https://github.com/CanDIG/omop_service

OHDSI, OMOP Common Data Model,

https://www.ohdsi.org/data-standardization/the-common-data-model/

CanDIG V2 Authentication and Authorization,

https://www.distributedgenomics.ca/posts/candigv2-aai/

Open Policy Agent (OPA), https://www.openpolicyagent.org/

ELIXIR, https://www.elixir-finland.org/en/aai-rems-2/

Workflow Execution Service (WES),

https://github.com/ga4gh/workflow-execution-service-schemas

CanCOGeN HostSeq, https://www.genomecanada.ca/en/cancogen/cancogen-hostseq

Digital Health & Discovery Platform (DHDP), https://www.dhdp.ca/

Marathon of Hope Cancer Centres, https://www.marathonofhopecancercentres.ca/

Supplementary Material References

1. Conley, R.B., Dickson, D., Zenklusen, J.C., Al Naber, J., Messner, D.A., Atasoy, A., Chaihorsky, L., Collyar, D., Compton, C., Ferguson, M., et al. (2017). Core Clinical Data Elements for Cancer Genomic Repositories: A Multi-stakeholder Consensus. Cell *171*, 982–986.

2. Amstutz, P., Crusoe, M.R., Tijanić, N., Chapman, B., Chilton, J., Heuer, M., Kartashov, A., Leehr, D., Ménager, H., Nedeljkovich, M., et al. (2016). Common Workflow Language, v1.0.

3. Linden, M., Prochazka, M., Lappalainen, I., Bucik, D., Vyskocil, P., Kuba, M., Silén, S., Belmann, P., Sczyrba, A., Newhouse, S., et al. (2018). Common ELIXIR Service for Researcher Authentication and Authorisation. F1000Res. *7*.

4. Sakimura, N., Bradley, J., Jones, M., de Medeiros, B., and Mortimore, C. (2014). OpenID Connect Core 1.0 incorporating errata set 1. The OpenID Foundation, specification.

5. Duchi, J.C., Jordan, M.I., and Wainwright, M.J. (2013). Local Privacy and Statistical Minimax Rates. In 2013 IEEE 54th Annual Symposium on Foundations of Computer Science, pp. 429–438.

6. Quinlan, J.R. (1986). Induction of decision trees. Mach. Learn. *1*, 81–106.

7. Kidd, K.K., Speed, W.C., Pakstis, A.J., Furtado, M.R., Fang, R., Madbouly, A., Maiers, M., Middha, M., Friedlaender, F.R., and Kidd, J.R. (2014). Progress toward an efficient panel of SNPs for ancestry inference. Forensic Sci. Int. Genet. *10*, 23–32.

8. Pleasance, E., Titmuss, E., Williamson, L., Kwan, H., Culibrk, L., Zhao, E.Y., Dixon, K., Fan, K., Bowlby, R., Jones, M.R., et al. (2020). Pan-cancer analysis of advanced patient tumors reveals interactions between therapy and genomic landscapes. Nature Cancer *1*, 452–468.

9. Clouthier, D.L., Lien, S.C., Yang, S.Y.C., Nguyen, L.T., Manem, V.S.K., Gray, D., Ryczko, M., Razak, A.R.A., Lewin, J., Lheureux, S., et al. (2019). An interim report on the investigator-initiated phase 2 study of pembrolizumab immunological response evaluation (INSPIRE). J Immunother Cancer *7*, 72.

10. Bratman, S.V., Yang, S.Y.C., Iafolla, M.A.J., Liu, Z., Hansen, A.R., Bedard, P.L., Lheureux, S., Spreafico, A., Razak, A.A., Shchegrova, S., et al. (2020). Personalized circulating tumor DNA analysis as a predictive biomarker in solid tumor patients treated with pembrolizumab. Nature Cancer *1*, 873–881.

11. Cindy Yang, S.Y., Lien, S.C., Wang, B.X., Clouthier, D.L., Hanna, Y., Cirlan, I., Zhu, K., Bruce, J.P., El Ghamrasni, S., Iafolla, M.A.J., et al. (2021). Pan-cancer analysis of longitudinal metastatic tumors reveals genomic alterations and immune landscape dynamics associated with pembrolizumab sensitivity. Nat. Commun. *12*, 5137.

12. Grover, S.A., Berman, J.N., Chan, J.A., Deyell, R.J., Eisenstat, D.D., Fernandez, C.V., Grundy, P.E., Hawkins, C., Irwin, M.S., Jabado, N., et al. (2020). Terry Fox PRecision Oncology For Young peopLE (PROFYLE): A Canadian precision medicine program for

children, adolescents and young adults with hard-to-treat cancer. Cancer Res *80*, 5413.

13. Fiume, M., Cupak, M., Keenan, S., Rambla, J., de la Torre, S., Dyke, S.O.M., Brookes, A.J., Carey, K., Lloyd, D., Goodhand, P., et al. (2019). Federated discovery and sharing of genomic data using Beacons. Nat. Biotechnol. *37*, 220–224.

14. Philippakis, A.A., Azzariti, D.R., Beltran, S., Brookes, A.J., Brownstein, C.A., Brudno, M., Brunner, H.G., Buske, O.J., Carey, K., Doll, C., et al. (2015). The Matchmaker Exchange: a platform for rare disease gene discovery. Hum. Mutat. *36*, 915–921.

15. Gaye, A., Marcon, Y., Isaeva, J., LaFlamme, P., Turner, A., Jones, E.M., Minion, J., Boyd, A.W., Newby, C.J., Nuotio, M.-L., et al. (2014). DataSHIELD: taking the analysis to the data, not the data to the analysis. Int. J. Epidemiol. *43*, 1929–1944.

16. Fernández-Orth, D., Lloret-Villas, A., and Rambla de Argila, J. (2019). European Genome-Phenome Archive (EGA) - Granular Solutions for the Next 10 Years. In 2019 IEEE 32nd International Symposium on Computer-Based Medical Systems (CBMS) (ieeexplore.ieee.org), pp. 4–6.

17. Raisaro, J.L., Choi, G., Pradervand, S., Colsenet, R., Jacquemont, N., Rosat, N., Mooser, V., and Hubaux, J. (2018). Protecting Privacy and Security of Genomic Data in i2b2 with Homomorphic Encryption and Differential Privacy. IEEE/ACM Trans. Comput. Biol. Bioinform. *15*, 1413–1426.
